# Supplementary material for: Using genetic variants to evaluate the causal effect of cholesterol lowering on head and neck cancer risk: A Mendelian randomization study
Source: PLoS Genet. 2021 Apr 22;17(4):e1009525. doi: 10.1371/journal.pgen.1009525 (PMC8096036; doi:10.1371/journal.pgen.1009525)
Supplement: S2 Table — (DOCX) [file pgen.1009525.s003.docx]

**S2 Tables.** Genetic correlation results for HMGCR, NPC1L1, CETP, PCSK9 and LDLR single nucleotide polymorphisms

| **HMGCR** | rs10066707 | rs2303152 | rs17238484 | rs5909 | rs12916 |
| --- | --- | --- | --- | --- | --- |
| rs10066707 | 1 | 0.068 | 0.266 | 0.106 | 0.31 |
| rs2303152 | 0.068 | 1 | 0.317 | 0.013 | 0.13 |
| rs17238484 | 0.266 | 0.317 | 1 | 0.04 | 0.378 |
| rs5909 | 0.106 | 0.013 | 0.04 | 1 | 0.213 |
| rs12916 | 0.31 | 0.13 | 0.378 | 0.213 | 1 |

| **NPC1L1** | rs10234070 | rs2073547 | rs217386 | rs7791240 | rs2300414 |
| --- | --- | --- | --- | --- | --- |
| rs10234070 | 1 | 0.198 | 0.048 | 0 | 0.002 |
| rs2073547 | 0.198 | 1 | 0.135 | 0.295 | 0.08 |
| rs217386 | 0.048 | 0.135 | 1 | 0.077 | 0.042 |
| rs7791240 | 0 | 0.295 | 0.077 | 1 | 0.36 |
| rs2300414 | 0.002 | 0.08 | 0.042 | 0.36 | 1 |

| **CETP** | **rs9989419** | **rs12708967** | **rs3764261** | **rs1800775** | **rs1864163** | **rs289714** |
| --- | --- | --- | --- | --- | --- | --- |
| **rs9989419** | 1 | 0.124 | 0.232 | 0.272 | 0.259 | 0.089 |
| **rs12708967** | 0.124 | 1 | 0.105 | 0.033 | 0.191 | 0.198 |
| **rs3764261** | 0.232 | 0.105 | 1 | 0.469 | 0.179 | 0.045 |
| **rs1800775** | 0.272 | 0.033 | 0.469 | 1 | 0.381 | 0.135 |
| **rs1864163** | 0.259 | 0.191 | 0.179 | 0.381 | 1 | 0.418 |
| **rs289714** | 0.089 | 0.198 | 0.045 | 0.135 | 0.418 | 1 |

| **PCSK9** | rs2479394 | rs11206510 | rs2479409 | rs10888897 | rs7552841 | rs562556 |
| --- | --- | --- | --- | --- | --- | --- |
| rs2479394 | 1 | 0.053 | 0.011 | 0.002 | 0.03 | 0.006 |
| rs11206510 | 0.053 | 1 | 0.067 | 0.068 | 0.018 | 0 |
| rs2479409 | 0.011 | 0.067 | 1 | 0.09 | 0 | 0.001 |
| rs10888897 | 0.002 | 0.068 | 0.09 | 1 | 0 | 0.047 |
| rs7552841 | 0.03 | 0.018 | 0 | 0 | 1 | 0.063 |
| rs562556 | 0.006 | 0 | 0.001 | 0.047 | 0.063 | 1 |

| **LDLR** | rs1122608 | rs6511720 | rs688 |
| --- | --- | --- | --- |
| rs1122608 | 1 | 0.202 | 0.084 |
| rs6511720 | 0.202 | 1 | 0.006 |
| rs688 | 0.084 | 0.006 | 1 |
