# Supplementary material for: Using genetic variants to evaluate the causal effect of cholesterol lowering on head and neck cancer risk: A Mendelian randomization study
Source: PLoS Genet. 2021 Apr 22;17(4):e1009525. doi: 10.1371/journal.pgen.1009525 (PMC8096036; doi:10.1371/journal.pgen.1009525)
Supplement: S3 Table — (DOCX) [file pgen.1009525.s004.docx]

**S3 Table.** Mendelian randomization results of genetically proxied inhibition of HMGCR, NPC1L1, CETP, PCSK9 and LDLR with risk of combined oral/ oropharyngeal cancer accounting for LD structure in GAME-ON

| **Outcome** | **Exposure** | **Method** | **Beta** | **se** | **OR** | **CIL** | **CIU** | ***P*-value** |
| --- | --- | --- | --- | --- | --- | --- | --- | --- |
| HNSCC | HMGCR | IVW | 0.0529 | 0.370 | 1.05 | 0.51 | 2.18 | 0.89 |
| HNSCC | HMGCR | MR Egger | 0.5254 | 1.344 | 1.69 | 0.12 | 23.54 | 0.70 |
| HNSCC | HMGCR | Weighted median | 0.1797 | 0.331 | 1.20 | 0.63 | 2.29 | 0.59 |
| HNSCC | NPC1L1 | IVW | -0.0068 | 0.595 | 0.99 | 0.31 | 3.19 | 0.99 |
| HNSCC | NPC1L1 | MR Egger | -1.5396 | 6.262 | 0.21 | 1.00E-06 | 4.59E+04 | 0.81 |
| HNSCC | NPC1L1 | Weighted median | -0.1059 | 0.583 | 0.90 | 0.29 | 2.82 | 0.86 |
| HNSCC | CETP | IVW | 0.3201 | 0.4943 | 1.38 | 0.52 | 3.63 | 0.52 |
| HNSCC | CETP | MR Egger | 0.2994 | 1.8661 | 1.35 | 0.03 | 52.30 | 0.87 |
| HNSCC | CETP | Weighted median | 0.2133 | 0.4271 | 1.24 | 0.54 | 2.86 | 0.62 |
| HNSCC | PCSK9 | IVW | 0.7086 | 0.275 | 2.03 | 1.18 | 3.48 | 0.01 |
| HNSCC | PCSK9 | MR Egger | 0.5135 | 1.137 | 1.67 | 0.18 | 15.53 | 0.65 |
| HNSCC | PCSK9 | Weighted median | 0.7934 | 0.318 | 2.21 | 1.18 | 4.13 | 0.01 |
| HNSCC | LDLR | IVW | -0.3696 | 0.216 | 0.69 | 0.45 | 1.06 | 0.09 |
| HNSCC | LDLR | MR Egger | -0.0518 | 0.372 | 0.95 | 0.46 | 1.97 | 0.89 |
| HNSCC | LDLR | Weighted median | -0.3417 | 0.216 | 0.71 | 0.47 | 1.09 | 0.11 |

Abbreviations: se, standard error; OR, odds ratio; CIL, lower confidence interval; CIU, upper confidence interval
