# Supplementary material for: Using genetic variants to evaluate the causal effect of cholesterol lowering on head and neck cancer risk: A Mendelian randomization study
Source: PLoS Genet. 2021 Apr 22;17(4):e1009525. doi: 10.1371/journal.pgen.1009525 (PMC8096036; doi:10.1371/journal.pgen.1009525)
Supplement: S4 Table — (DOCX) [file pgen.1009525.s005.docx]

**S4 Table.** Assessing weak instrument bias (F-statistic) and proportion of variance in the phenotype (R^2^) explained by the genetic instruments

|  | R^2^ | F-statistic |
| --- | --- | --- |
| **HMGCR** | 0.003 | 111 |
| **NPC1L1** | 0.001 | 51 |
| **CETP** | 0.003 | 68 |
| **PCSK9** | 0.005 | 140 |
| **LDLR** | 0.008 | 508 |
| **HDL-C** | 0.05 | 108 |
| **LDL-C** | 0.06 | 140 |
| **Total cholesterol** | 0.06 | 132 |
| **Total triglycerides** | 0.04 | 163 |
| **Apolipoprotein A** | 0.004 | 83 |
| **Apolipoprotein B** | 0.007 | 72 |
