# Supplementary material for: Using genetic variants to evaluate the causal effect of cholesterol lowering on head and neck cancer risk: A Mendelian randomization study
Source: PLoS Genet. 2021 Apr 22;17(4):e1009525. doi: 10.1371/journal.pgen.1009525 (PMC8096036; doi:10.1371/journal.pgen.1009525)
Supplement: S5 Table — Abbreviations: Q, Q-statistic; df, degrees of freedom; P, p-value. (DOCX) [file pgen.1009525.s006.docx]

**S5 Table.** Assessing heterogeneity of single nucleotide polymorphism effect estimates in inverse-variance weighted (IVW) and MR Egger regression for primary analysis

| **Exposure** | **Exposure dataset** | **Q IVW** | **df** | **P** | **Q MR Egger** | **df** | **P** |
| --- | --- | --- | --- | --- | --- | --- | --- |
| HMGCR | GLGC^24^ | 2.75 | 4 | 0.60 | 2.73 | 3 | 0.44 |
| NPC1L1 | GLGC^24^ | 0.76 | 4 | 0.94 | 0.52 | 3 | 0.92 |
| CETP | GLGC^24^ | 1.99 | 5 | 0.85 | 1.92 | 4 | 0.75 |
| PCSK9 | GLGC^24^ | 4.61 | 5 | 0.47 | 4.60 | 4 | 0.33 |
| LDLR | GLGC^24^ | 2.33 | 2 | 0.31 | 1.18 | 1 | 0.28 |

Abbreviations: Q, Q-statistic; df, degrees of freedom; P, p-value.
