# Supplementary material for: Using genetic variants to evaluate the causal effect of cholesterol lowering on head and neck cancer risk: A Mendelian randomization study
Source: PLoS Genet. 2021 Apr 22;17(4):e1009525. doi: 10.1371/journal.pgen.1009525 (PMC8096036; doi:10.1371/journal.pgen.1009525)
Supplement: S6 Table — Abbreviations: Q, Q-statistic; df, degrees of freedom; P, p-value. (DOCX) [file pgen.1009525.s007.docx]

**S6 Table.** Assessing heterogeneity of single nucleotide polymorphism effect estimates in inverse-variance weighted (IVW) and MR Egger regression for secondary analysis

| **Exposure** | **Exposure dataset** | **Q IVW** | **df** | **P** | **Q MR Egger** | **df** | **P** |
| --- | --- | --- | --- | --- | --- | --- | --- |
| HDL-C | GLGC^24^ | 115.65 | 84 | 0.01 | 115.56 | 83 | 0.01 |
| LDL-C | GLGC^24^ | 86.91 | 76 | 0.18 | 86.87 | 75 | 0.16 |
| Total cholesterol | GLGC^24^ | 92.58 | 81 | 0.18 | 92.22 | 80 | 0.17 |
| Total triglycerides | GLGC^24^ | 59.01 | 53 | 0.27 | 58.41 | 52 | 0.25 |
| Apolipoprotein A | 14 studies (Kettunen et al.)^33^ | 7.39 | 8 | 0.50 | 6.65 | 7 | 0.47 |
| Apolipoprotein B | 14 studies (Kettunen et al.)^33^ | 18.11 | 13 | 0.15 | 18.05 | 12 | 0.11 |

Abbreviations: Q, Q-statistic; df, degrees of freedom; P, p-value.
