# Supplementary material for: Using genetic variants to evaluate the causal effect of cholesterol lowering on head and neck cancer risk: A Mendelian randomization study
Source: PLoS Genet. 2021 Apr 22;17(4):e1009525. doi: 10.1371/journal.pgen.1009525 (PMC8096036; doi:10.1371/journal.pgen.1009525)
Supplement: S7 Table — Abbreviations: UVMR, univariable Mendelian randomization; SE, standard error; P, p-value. (DOCX) [file pgen.1009525.s008.docx]

**S7 Table.** Assessing directional pleiotropy through MR Egger intercept for primary analysis

| **Exposure** | **Exposure dataset** | **N SNPs** | **Estimate** | **SE** | **P** |
| --- | --- | --- | --- | --- | --- |
| HMGCR | GLGC^24^ | 5 | 0.011 | 0.076 | 0.89 |
| NPC1L1 | GLGC^24^ | 5 | -0.049 | 0.099 | 0.65 |
| CETP | GLGC^22^ | 6 | -0.016 | 0.062 | 0.81 |
| PCSK9 | GLGC^24^ | 6 | -0.006 | 0.059 | 0.93 |
| LDLR | GLGC^24^ | 3 | 0.035 | 0.035 | 0.51 |

Abbreviations: UVMR, univariable Mendelian randomization; SE, standard error; P, p-value.
