# Supplementary material for: Using genetic variants to evaluate the causal effect of cholesterol lowering on head and neck cancer risk: A Mendelian randomization study
Source: PLoS Genet. 2021 Apr 22;17(4):e1009525. doi: 10.1371/journal.pgen.1009525 (PMC8096036; doi:10.1371/journal.pgen.1009525)
Supplement: S8 Table — Abbreviations: UVMR, univariable Mendelian randomization; SE, standard error; P, p-value. (DOCX) [file pgen.1009525.s009.docx]

**S8 Table.** Assessing directional pleiotropy through MR Egger intercept for secondary analysis

| **Exposure** | **Exposure dataset** | **N SNPs** | **Estimate** | **SE** | **P** |
| --- | --- | --- | --- | --- | --- |
| LDL-C | GLGC^24^ | 77 | 0.001 | 0.006 | 0.86 |
| HDL-C | GLGC^24^ | 85 | -0.002 | 0.007 | 0.80 |
| Total cholesterol | GLGC^24^ | 82 | -0.003 | 0.006 | 0.58 |
| Total triglycerides | GLGC^24^ | 54 | -0.005 | 0.007 | 0.47 |
| Apolipoprotein A | 14 studies (Kettunen et al.)^33^ | 9 | 0.029 | 0.033 | 0.42 |
| Apolipoprotein B | 14 studies (Kettunen et al.)^33^ | 14 | -0.004 | 0.019 | 0.85 |

Abbreviations: UVMR, univariable Mendelian randomization; SE, standard error; P, p-value.
