# Supplementary material for: Using genetic variants to evaluate the causal effect of cholesterol lowering on head and neck cancer risk: A Mendelian randomization study
Source: PLoS Genet. 2021 Apr 22;17(4):e1009525. doi: 10.1371/journal.pgen.1009525 (PMC8096036; doi:10.1371/journal.pgen.1009525)
Supplement: S9 Table — Abbreviations: RSSobs, residual sum of squares observations. (DOCX) [file pgen.1009525.s010.docx]

**S9 Table.** MR-PRESSO results for HMGCR, NPC1L1, CETP, PCSK9, LDLR and other lipid trait SNPs on combined oral/ oropharyngeal cancer

| **Outcome** | **Exposure** | **RSSobs** | **P-value** |
| --- | --- | --- | --- |
| HNSCC | HMGCR | 3.67 | 6.91E-01 |
| HNSCC | NPC1L1 | 1.06 | 9.58E-01 |
| HNSCC | CETP | 2.85 | 8.44 E-01 |
| HNSCC | PCSK9 | 6.52 | 4.84E-01 |
| HNSCC | LDLR | NA (insufficient SNPs) | NA (insufficient SNPs) |
| HNSCC | LDL-C | 90.49 | 0.16 |
| HNSCC | HDL-C | 117.57 | 0.01 |
| HNSCC | Total cholesterol | 95.11 | 0.181 |
| HNSCC | Total triglycerides | 61.08 | 0.28 |
| HNSCC | Apolipoprotein A | 8.57 | 0.56 |
| HNSCC | Apolipoprotein B | 21.82 | 0.13 |

Abbreviations: RSSobs, residual sum of squares observations.
