# Supplementary material for: Using genetic variants to evaluate the causal effect of cholesterol lowering on head and neck cancer risk: A Mendelian randomization study
Source: PLoS Genet. 2021 Apr 22;17(4):e1009525. doi: 10.1371/journal.pgen.1009525 (PMC8096036; doi:10.1371/journal.pgen.1009525)
Supplement: S10 Table — Abbreviations: I2, I-squared statistic. (DOCX) [file pgen.1009525.s011.docx]

**S10 Table.** Assessing violation of the “NO Measurement Error” (NOME) assumption for instruments used in MR-Egger regression

| **Exposure** | **Exposure dataset** | **I^2^ unweighted** | **I^2^ weighted** |
| --- | --- | --- | --- |
| HMGCR | GLGC^24^ | 0.78 | 0.93 |
| NPC1L1 | GLGC^24^ | 0.46 | 0.86 |
| CETP | GLGC^22^ | 0.67 | 0.86 |
| PCSK9 | GLGC^24^ | 0.99 | 0.98 |
| LDLR | GLGC^24^ | 0.99 | 0.99 |
| LDL-C | GLGC^24^ | 0.99 | 0.98 |
| HDL-C | GLGC^24^ | 0.97 | 0.97 |
| Total cholesterol | GLGC^24^ | 0.98 | 0.98 |
| Total triglycerides | GLGC^24^ | 0.99 | 0.98 |
| Apolipoprotein A | 14 studies (Kettunen et al.)^33^ | 0.92 | 0.95 |
| Apolipoprotein B | 14 studies (Kettunen et al.)^33^ | 0.95 | 0.93 |

Abbreviations: I^2^, I-squared statistic.
