# Supplementary material for: Using genetic variants to evaluate the causal effect of cholesterol lowering on head and neck cancer risk: A Mendelian randomization study
Source: PLoS Genet. 2021 Apr 22;17(4):e1009525. doi: 10.1371/journal.pgen.1009525 (PMC8096036; doi:10.1371/journal.pgen.1009525)
Supplement: S11 Table — Abbreviations: OR, odds ratio; CIL, lower confidence interval; CIU, upper confidence interval; P, p-value. (DOCX) [file pgen.1009525.s012.docx]

**S11 Table.** SIMEX correction MR Egger regression results for HMGCR, NPC1L1 and CETP (where I^2^ <0.90)

| **Outcome** | **Exposure** | **OR** | **CIL** | **CIU** | **P** |
| --- | --- | --- | --- | --- | --- |
| HNSCC | HMGCR | 0.60 | 0.03 | 12.20 | 0.76 |
| HNSCC | NPC1L1 | 7.77 | 0.19 | 321.65 | 0.36 |
| HNSCC | CETP | 1.82 | 0.03 | 120.28 | 0.79 |

Abbreviations: OR, odds ratio; CIL, lower confidence interval; CIU, upper confidence interval; P, p-value.
