# Supplementary material for: Using genetic variants to evaluate the causal effect of cholesterol lowering on head and neck cancer risk: A Mendelian randomization study
Source: PLoS Genet. 2021 Apr 22;17(4):e1009525. doi: 10.1371/journal.pgen.1009525 (PMC8096036; doi:10.1371/journal.pgen.1009525)
Supplement: S12 Table — (DOCX) [file pgen.1009525.s013.docx]

**S12 Table.** Colocalisation results for PCSK9 and LDLR with oral and oropharyngeal cancer combined and LDL-C.

| **PCSK9** | **N SNPs** | 1481 |
| --- | --- | --- |
|  | **H0: neither trait has a genetic association in the region** | 2.66E-135 |
|  | **H1: only trait 1 has a genetic association in the region** | 0.856 |
|  | **H2: only trait 2 has a genetic association in the region** | 2.76E-136 |
|  | **H3: both traits are associated, but with different causal variants** | 0.089 |
|  | **H4: both traits are associated and share a single causal variant** | 0.055 |

| **LDLR** | **N SNPs** | 815 |
| --- | --- | --- |
|  | **H0: neither trait has a genetic association in the region** | 9.61E-255 |
|  | **H1: only trait 1 has a genetic association in the region** | 0.946 |
|  | **H2: only trait 2 has a genetic association in the region** | 2.91E-256 |
|  | **H3: both traits are associated, but with different causal variants** | 0.029 |
|  | **H4: both traits are associated and share a single causal variant** | 0.026 |
