# Supplementary material for: Using genetic variants to evaluate the causal effect of cholesterol lowering on head and neck cancer risk: A Mendelian randomization study
Source: PLoS Genet. 2021 Apr 22;17(4):e1009525. doi: 10.1371/journal.pgen.1009525 (PMC8096036; doi:10.1371/journal.pgen.1009525)
Supplement: S13 Table — Abbreviations: IVW, inverse variance weighted; OR, odds ratio; CI, confidence intervals; P, p-value. OR represents the exponential change in odds of oral/ oropharyngeal squamous cell carcinoma per genetically-proxied inhibition of drug target equivalent to a 1 mmol/L decrease in LDL-C. (DOCX) [file pgen.1009525.s014.docx]

**S13 Table.** Mendelian randomization results of genetically proxied inhibition of HMGCR, NPC1L1, CETP, PCSK9 and LDLR with risk of oral and oropharyngeal cancer including sensitivity analyses in UK Biobank

|  |  | | | | **IVW** | | **Weighted median** | | **Weighted mode** | | **MR-Egger** | |
| --- | --- | --- | --- | --- | --- | --- | --- | --- | --- | --- | --- | --- |
|  | **Outcome** | **Exposure/**  **Outcome**  **dataset** | **Outcome N** | **Number of SNPs** | **OR (95%CI)** | **P** | **OR (95%CI)** | **P** | **OR (95%CI)** | **P** | **OR (95%CI)** | **P** |
| HMGCR | Oral/ Oropharyngeal cancer | UK Biobank/ GLGC | 839 | 5 | 0.55 (0.21, 1.46) | 0.23 | 0.59 (0.18, 1.89) | 0.38 | 0.57 (0.14, 2.31) | 0.46 | 3.76 (0.01, 1145.91) | 0.67 |
|  | Oral cancer | UK Biobank/ GLGC | 357 | 5 | 0.24 (0.05, 1.09) | 0.06 | 0.48 (0.08, 3.03) | 0.44 | 0.49 (0.05, 4.33) | 0.55 | 31.78 (0.00, 203203.76) | 0.48 |
|  | Oropharyngeal cancer | UK Biobank/ GLGC | 494 | 5 | 1.01 (0.28, 3.63) | 0.99 | 0.74 (0.15, 3.67) | 0.71 | 0.75 (0.13, 4.33) | 0.76 | 0.52 (0.00, 890.93) | 0.87 |
| NPC1L1 | Oral/ Oropharyngeal cancer | UK Biobank/ GLGC | 839 | 5 | 1.14 (0.05, 24.82) | 0.94 | 2.54 (0.14, 45.32) | 0.52 | 3.79 (0.05, 284.57) | 0.58 | 0.02 (0.00, 46457078.93) | 0.76 |
|  | Oral cancer | UK Biobank/ GLGC | 357 | 5 | 2.91 (0.03, 277.95) | 0.65 | 6.99 (0.09, 554.04) | 0.38 | 22.20 (0.02, 27423.14) | 0.44 | 0.00 (0.00, 67338766.60) | 0.51 |
|  | Oropharyngeal cancer | UK Biobank/ GLGC | 494 | 5 | 0.50 (0.04, 5.88) | 0.58 | 1.58 (0.08, 32.81) | 0.77 | 2.72 (0.10, 74.56) | 0.59 | 4.39 (0.00, 39822777.00) | 0.87 |
| CETP | Oral/ Oropharyngeal cancer | UK Biobank/ GLGC | 839 | 5 | 2.66 (0.83, 8.59) | 0.10 | 2.19 (0.49, 9.71) | 0.30 | 1.74 (0.24, 12.48) | 0.60 | 9.07 (0.02, 4195.65) | 0.51 |
|  | Oral cancer | UK Biobank/ GLGC | 357 | 6 | 6.84 (1.14, 41.13) | 0.04 | 11.21 (1.16, 108.63) | 0.04 | 15.89 (0.76, 330.89) | 0.12 | 5.93 (0.00, 71920.27) | 0.72 |
|  | Oropharyngeal cancer | UK Biobank/ GLGC | 494 | 6 | 0.99 (0.21, 4.54) | 0.99 | 1.29 (0.19, 8.76) | 0.80 | 1.49 (0.14, 15.60) | 0.75 | 5.31 (0.00, 15730.94) | 0.70 |
| PCSK9 | Oral/ Oropharyngeal cancer | UK Biobank/ GLGC | 839 | 5 | 1.17 (0.50, 2.69) | 0.72 | 1.04 (0.37, 2.97) | 0.94 | 1.05 (0.24, 4.57) | 0.95 | 0.15 (0.00, 8.67) | 0.40 |
|  | Oral cancer | UK Biobank/ GLGC | 357 | 6 | 0.70 (0.19, 2.53) | 0.59 | 0.53 (0.09, 2.94) | 0.46 | 0.24 (0.01, 4.20) | 0.36 | 0.75 (0.00, 374.07) | 0.93 |
|  | Oropharyngeal cancer | UK Biobank/ GLGC | 494 | 6 | 1.43 (0.48, 4.26) | 0.52 | 1.33 (0.34, 5.19) | 0.68 | 0.80 (0.11, 5.81) | 0.83 | 0.07 (0.00, 13.21) | 0.36 |
| LDLR | Oral/ Oropharyngeal cancer | UK Biobank/ GLGC | 839 | 5 | 0.91 (0.44, 1.90) | 0.80 | 0.93 (0.43, 2.00) | 0.86 | 0.94 (0.41, 2.16) | 0.91 | 1.01 (0.27, 3.73) | 0.99 |
|  | Oral cancer | UK Biobank/ GLGC | 357 | 3 | 1.55 (0.50, 4.78) | 0.44 | 1.67 (0.51, 5.43) | 0.40 | 2.04 (0.53, 7.82) | 0.41 | 4.03 (0.55, 29.83) | 0.40 |
|  | Oropharyngeal cancer | UK Biobank/ GLGC | 494 | 3 | 0.54 (0.21, 1.39) | 0.20 | 0.47 (0.17, 1.31) | 0.15 | 0.47 (0.16, 1.37) | 0.30 | 0.35 (0.06, 1.92) | 0.44 |

Abbreviations: IVW, inverse variance weighted; OR, odds ratio; CI, confidence intervals; P, p-value.

OR represents the exponential change in odds of oral/ oropharyngeal squamous cell carcinoma per genetically proxied inhibition of drug target equivalent to a 1 mmol/L decrease in LDL-C.
