# Supplementary figures and images for: Using genetic variants to evaluate the causal effect of cholesterol lowering on head and neck cancer risk: A Mendelian randomization study
Source: PLoS Genet. 2021 Apr 22;17(4):e1009525. doi: 10.1371/journal.pgen.1009525 (PMC8096036; doi:10.1371/journal.pgen.1009525)

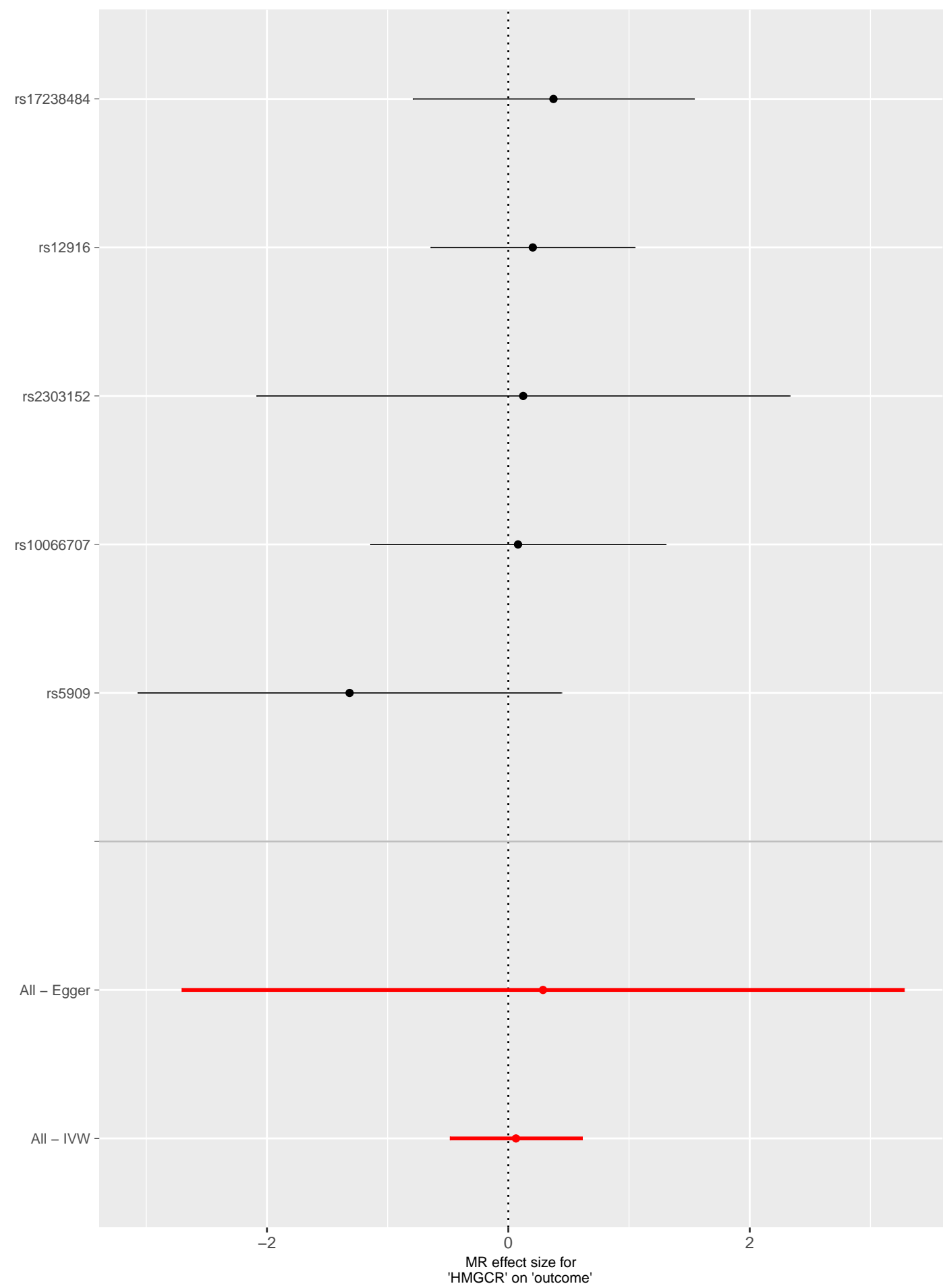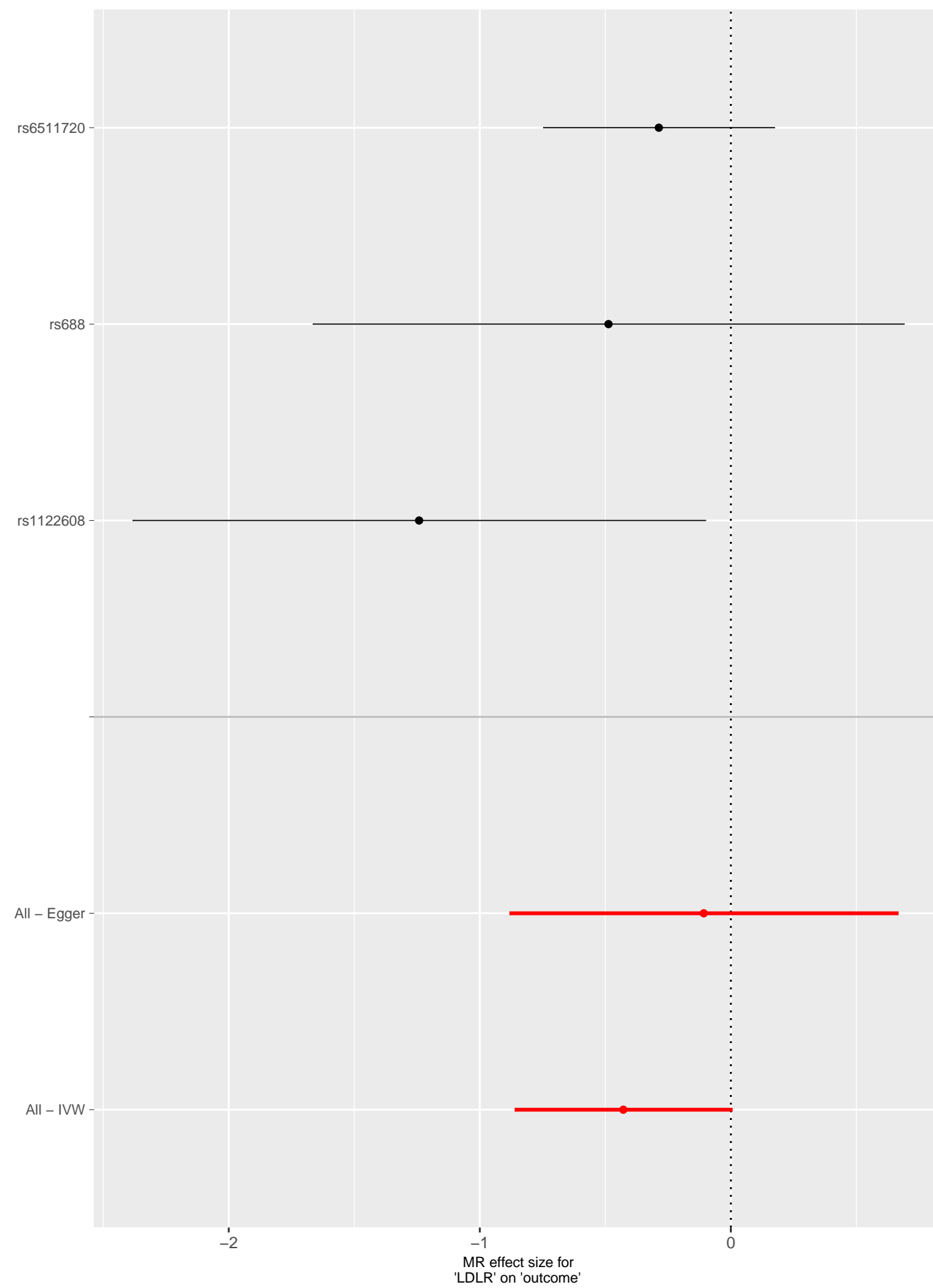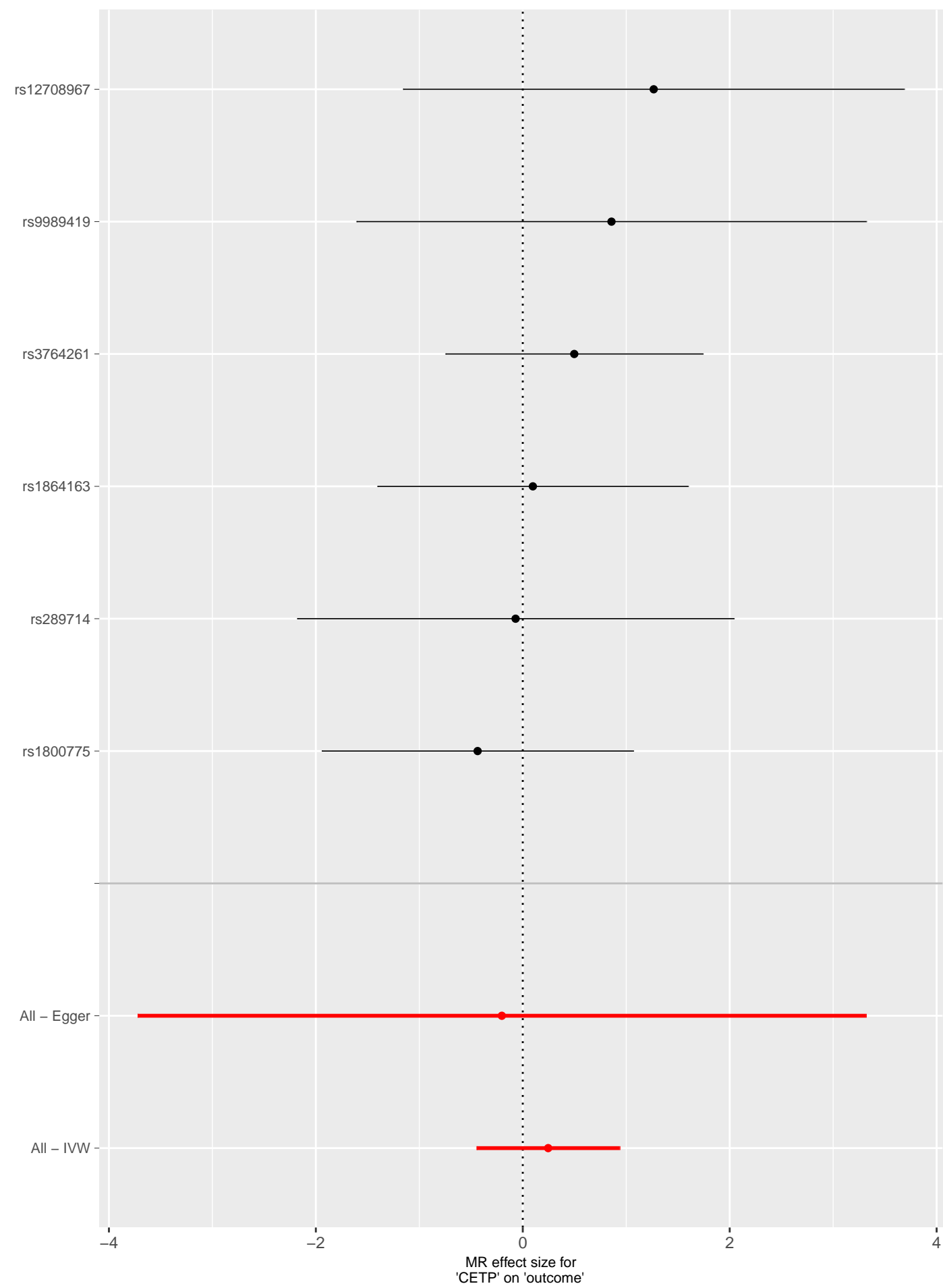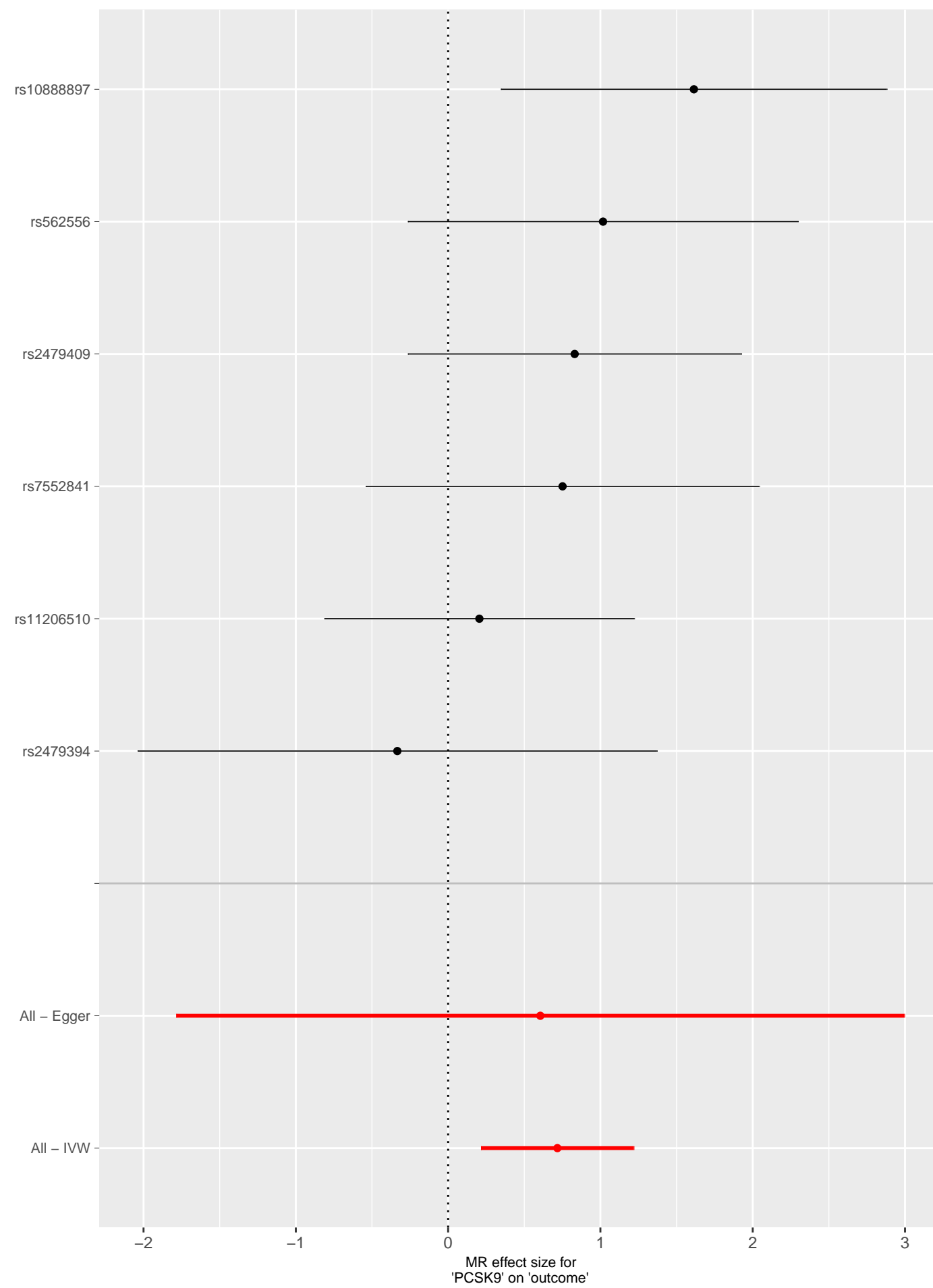

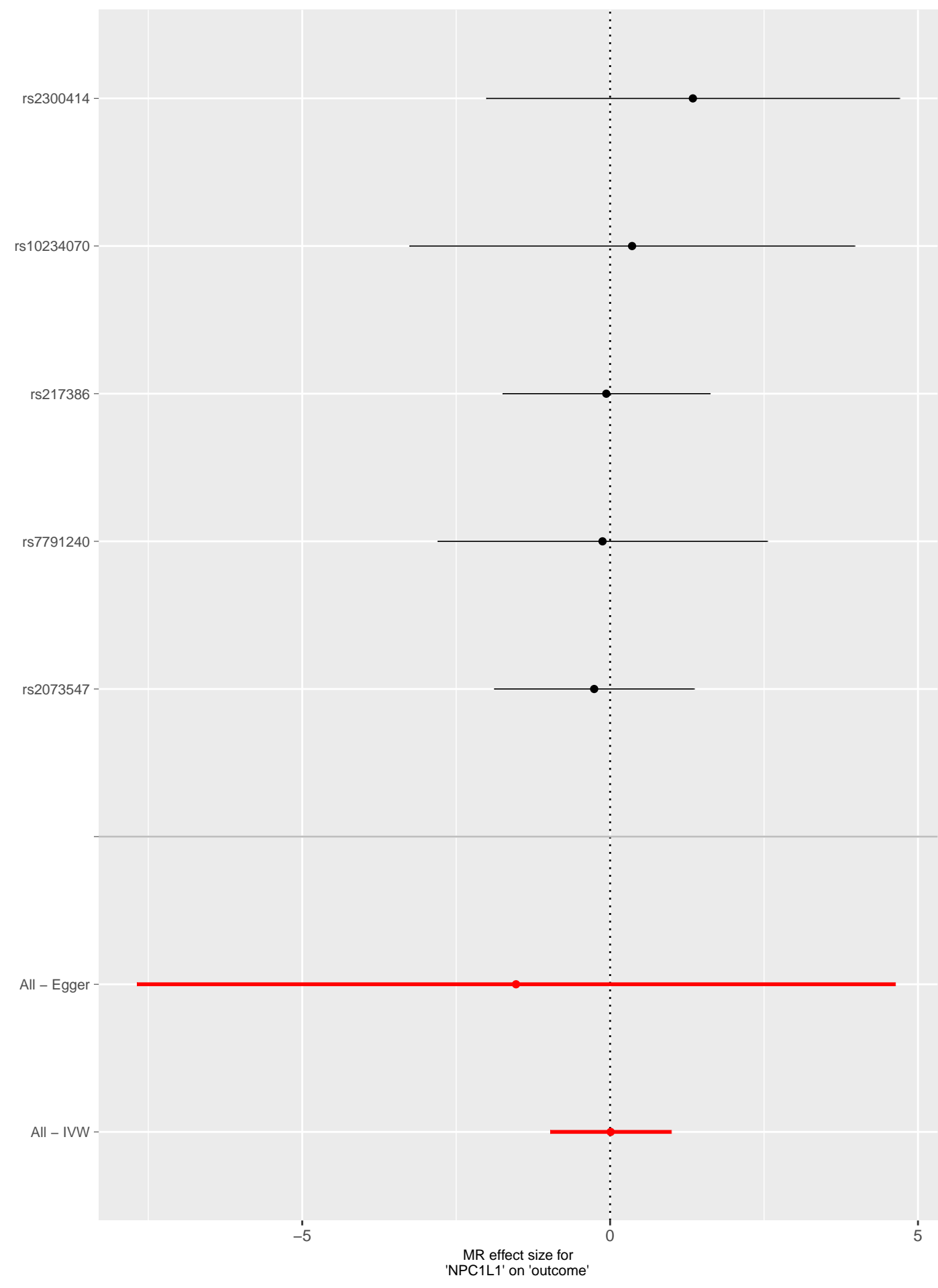

Supplement: S1 Fig — Effect estimates on oral and oropharyngeal cancer are reported on the log odds scale. (PDF) [file pgen.1009525.s015.pdf]

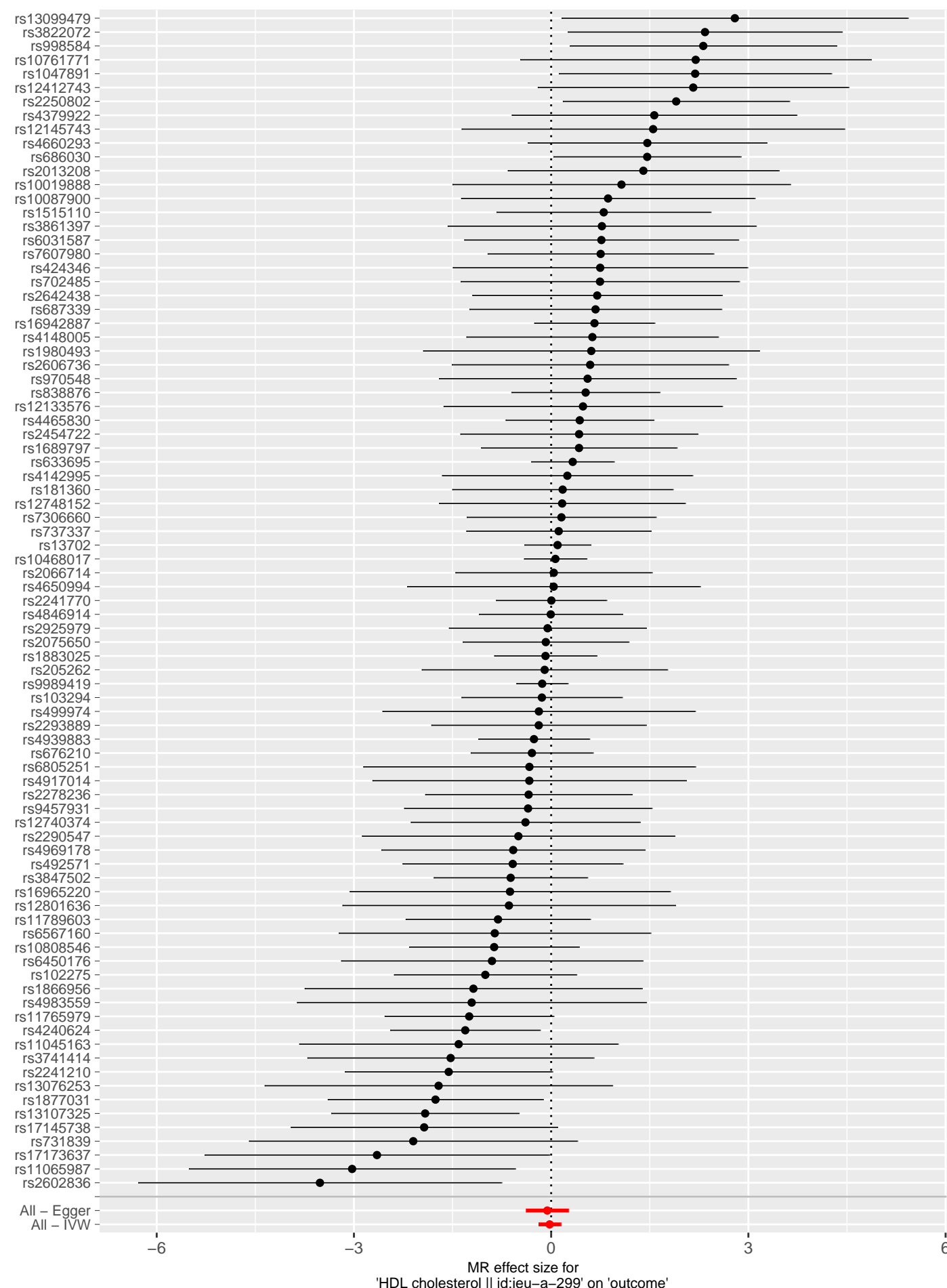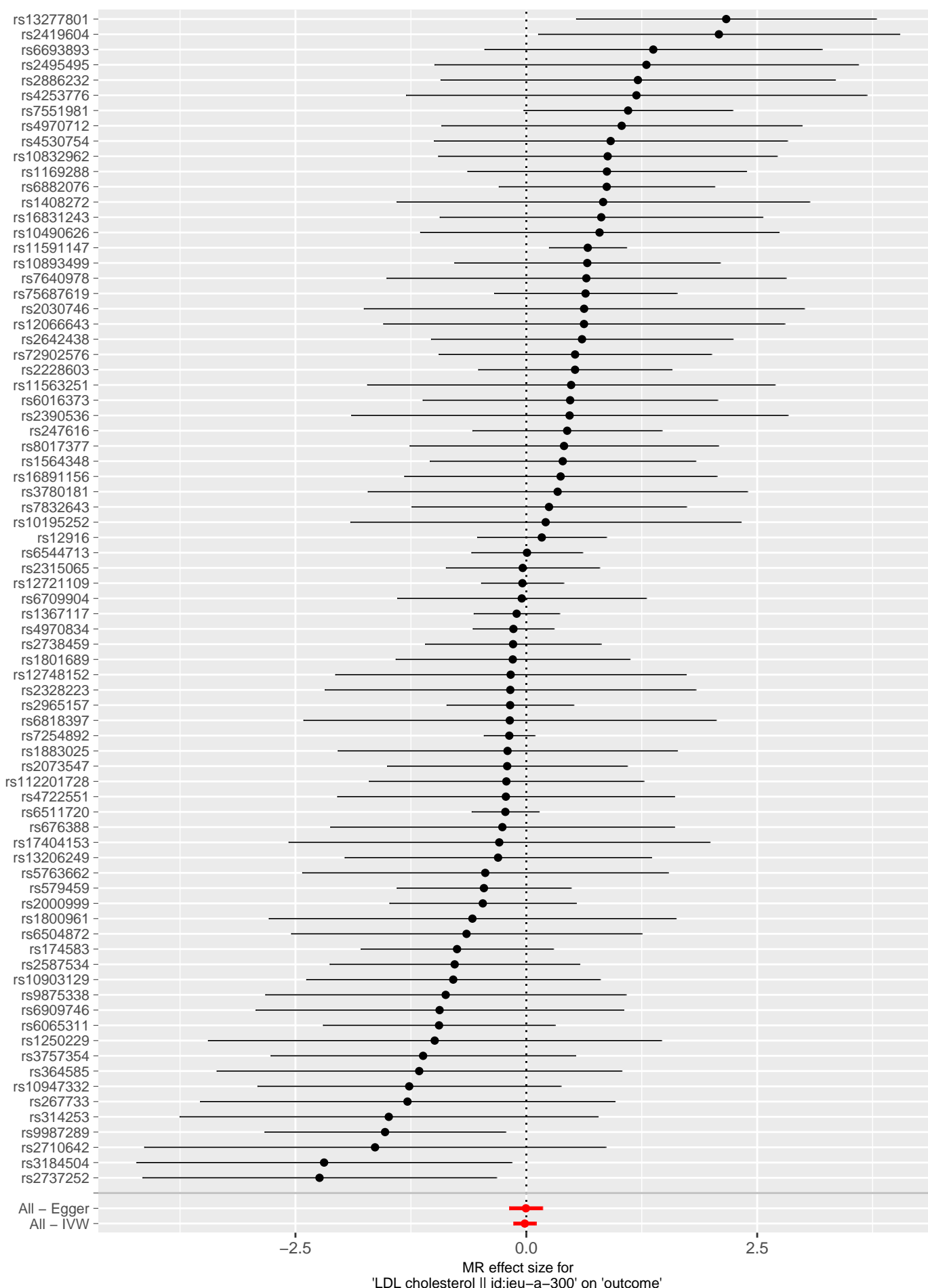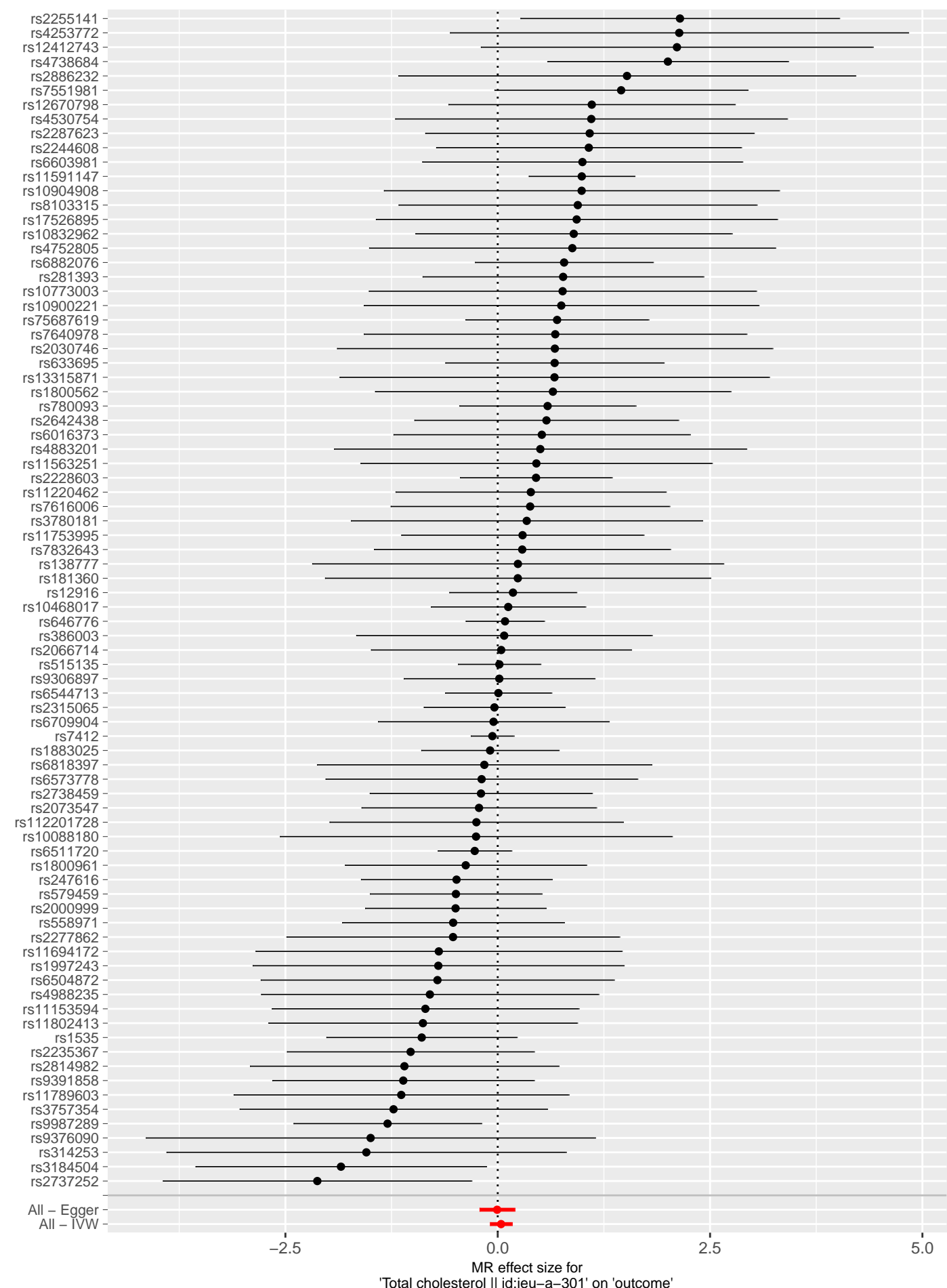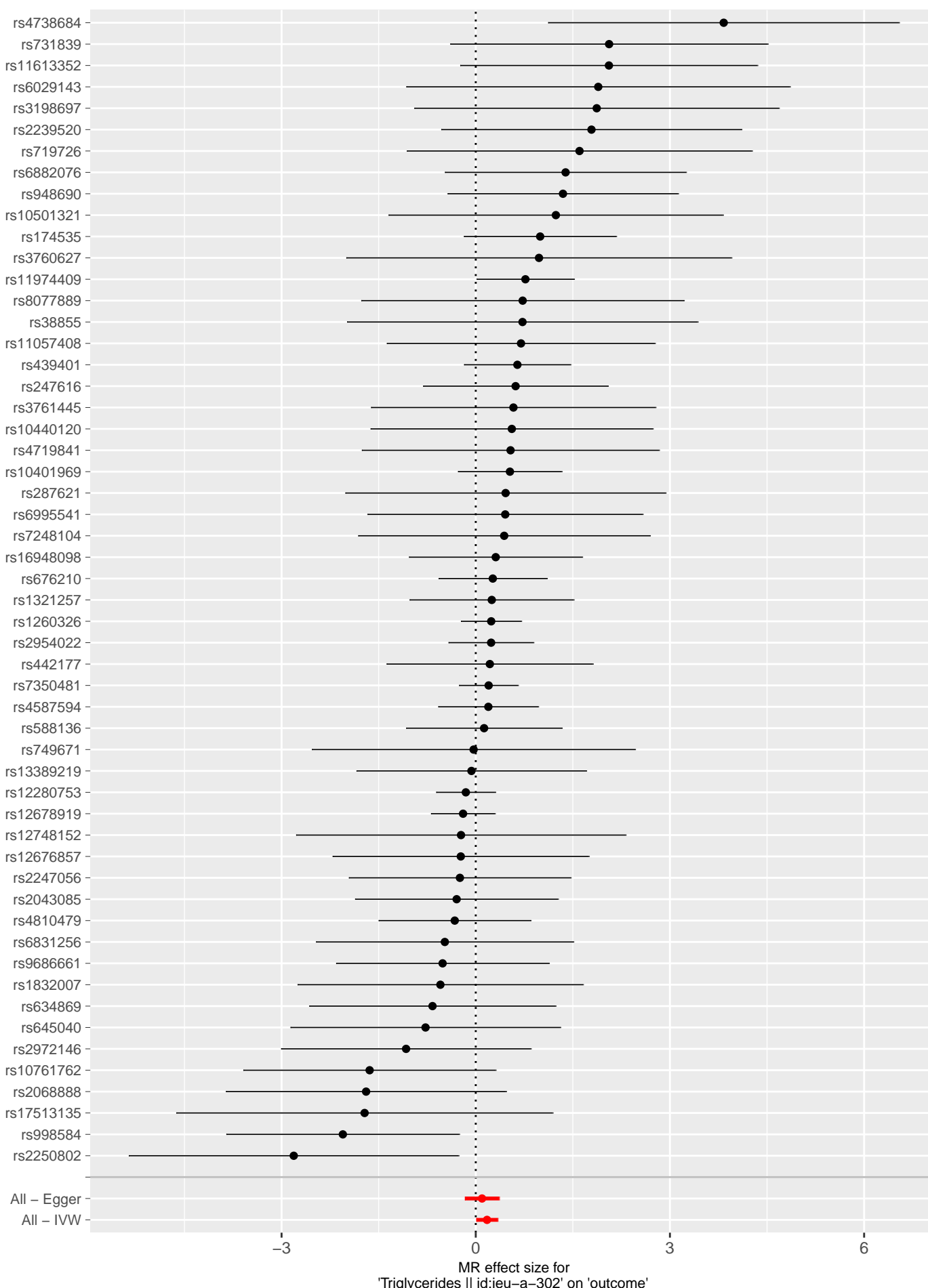

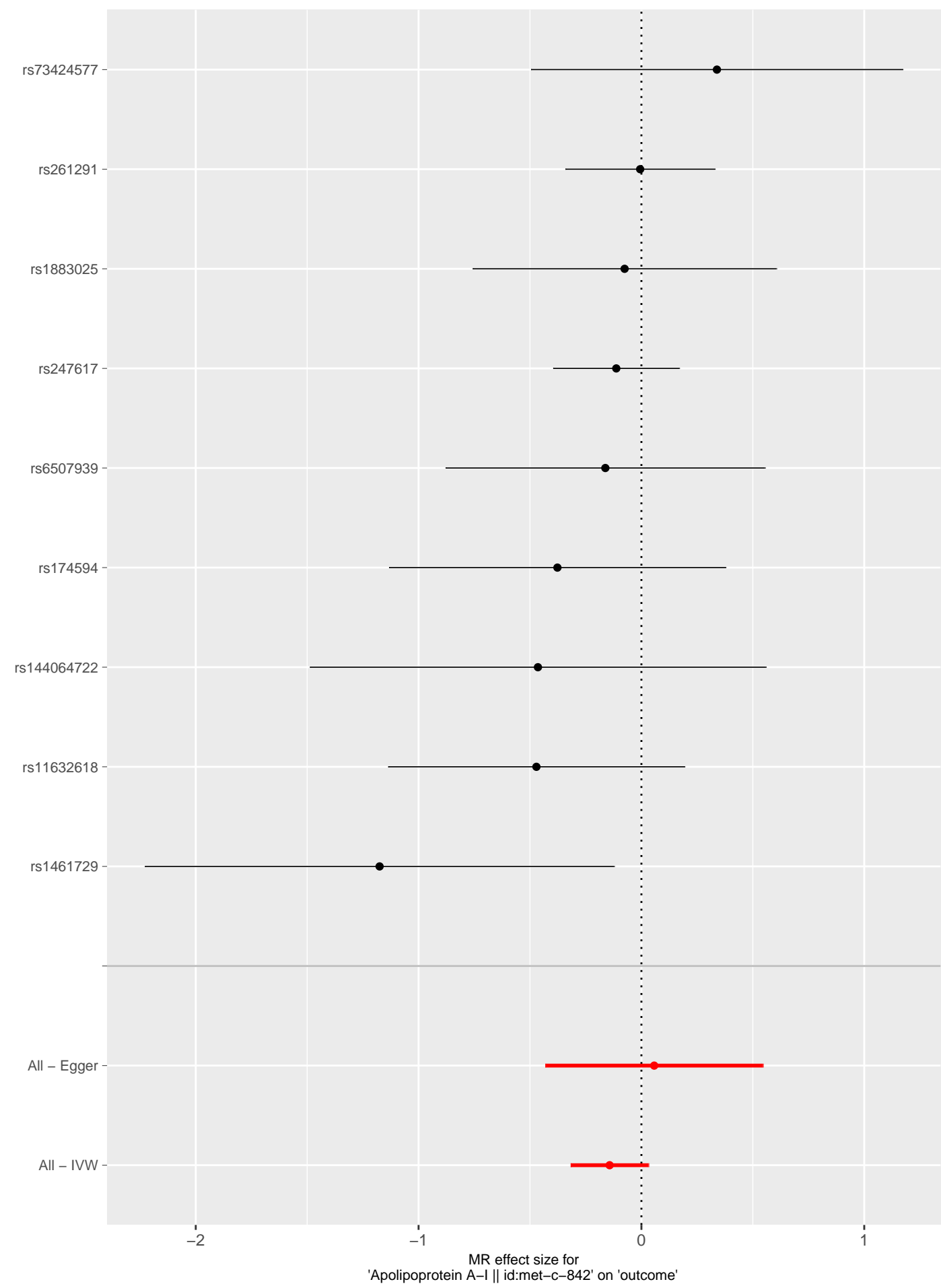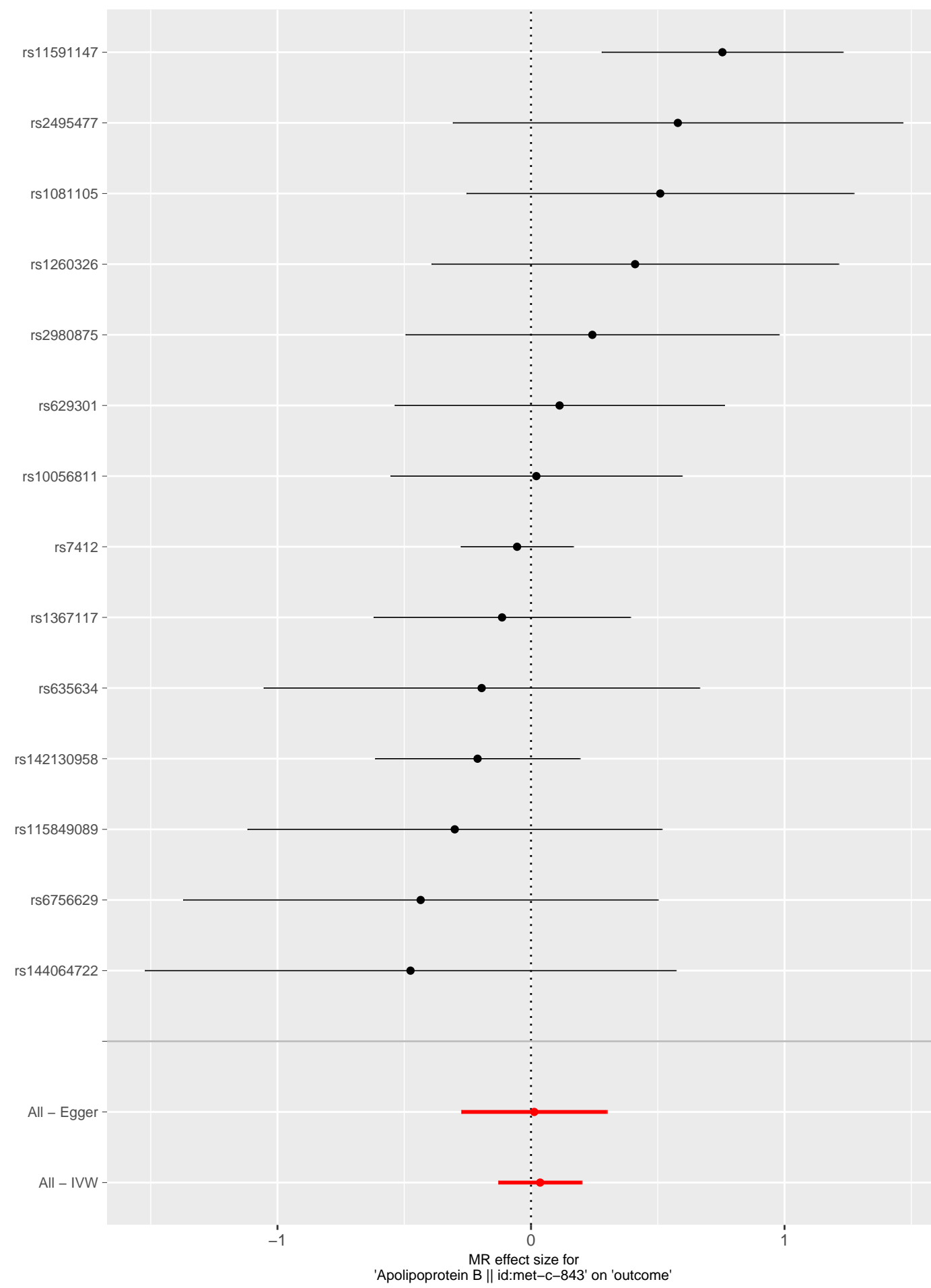

Supplement: S2 Fig — Effect estimates on oral and oropharyngeal cancer are reported on the log odds scale. (PDF) [file pgen.1009525.s016.pdf]

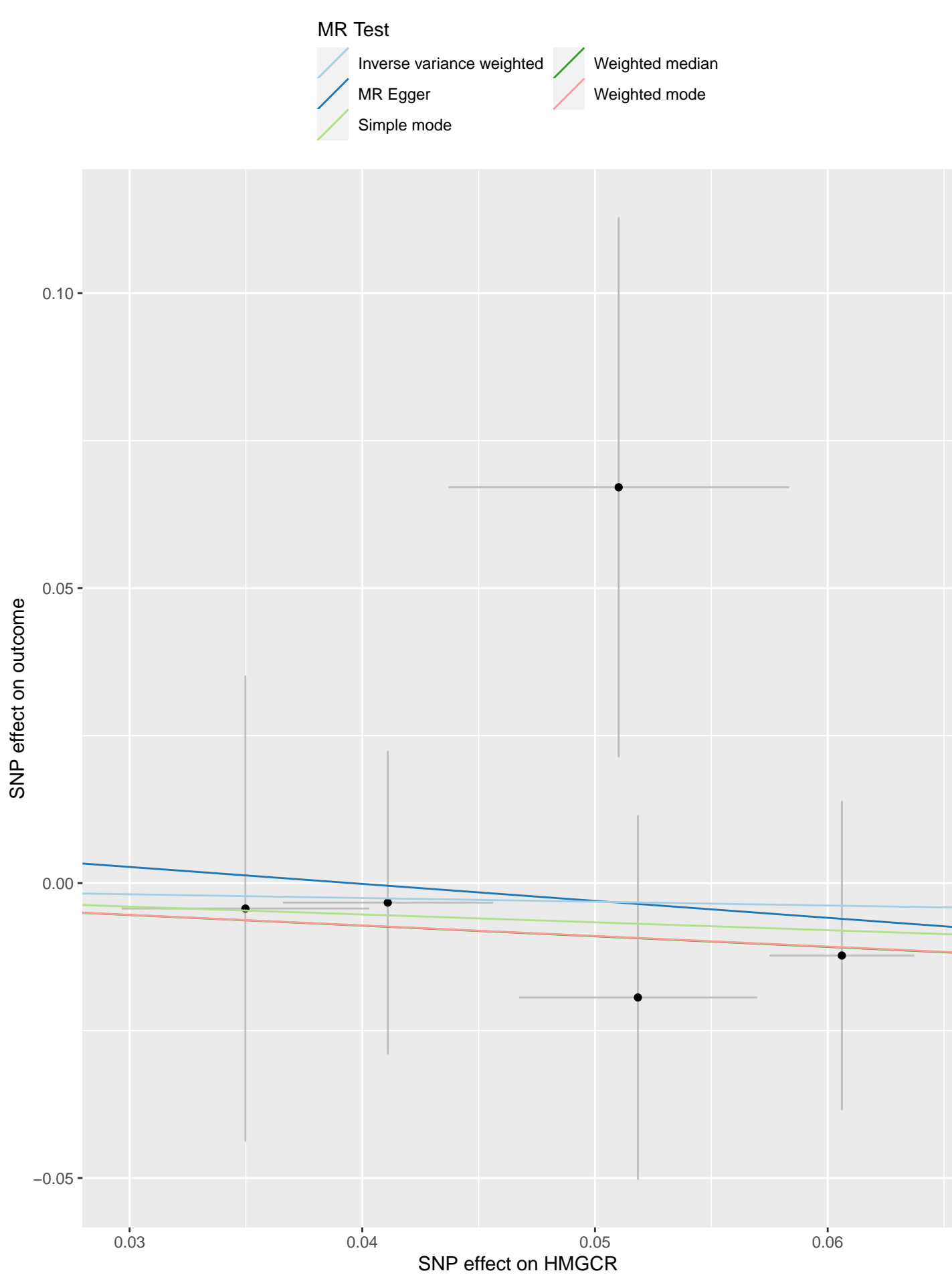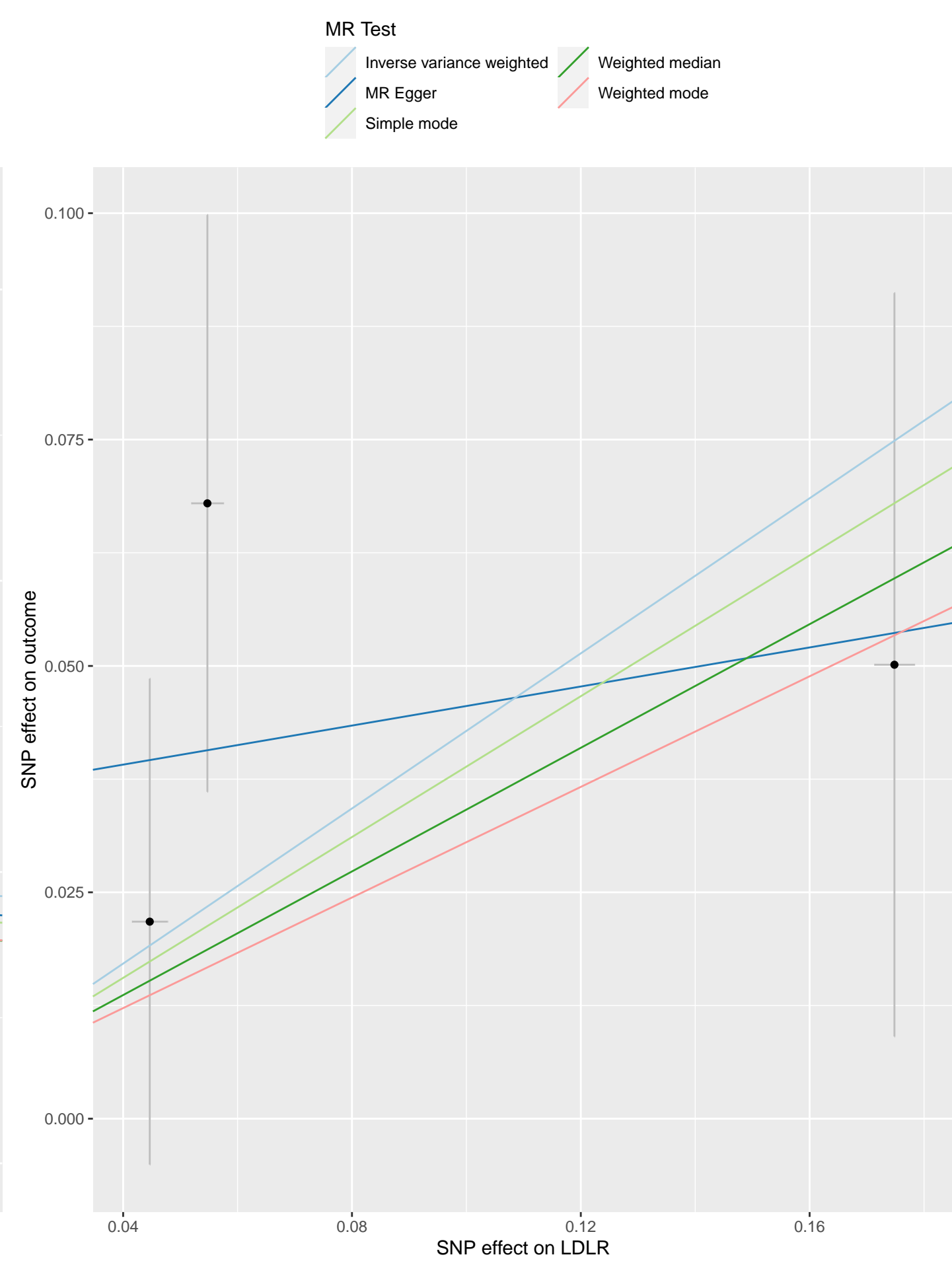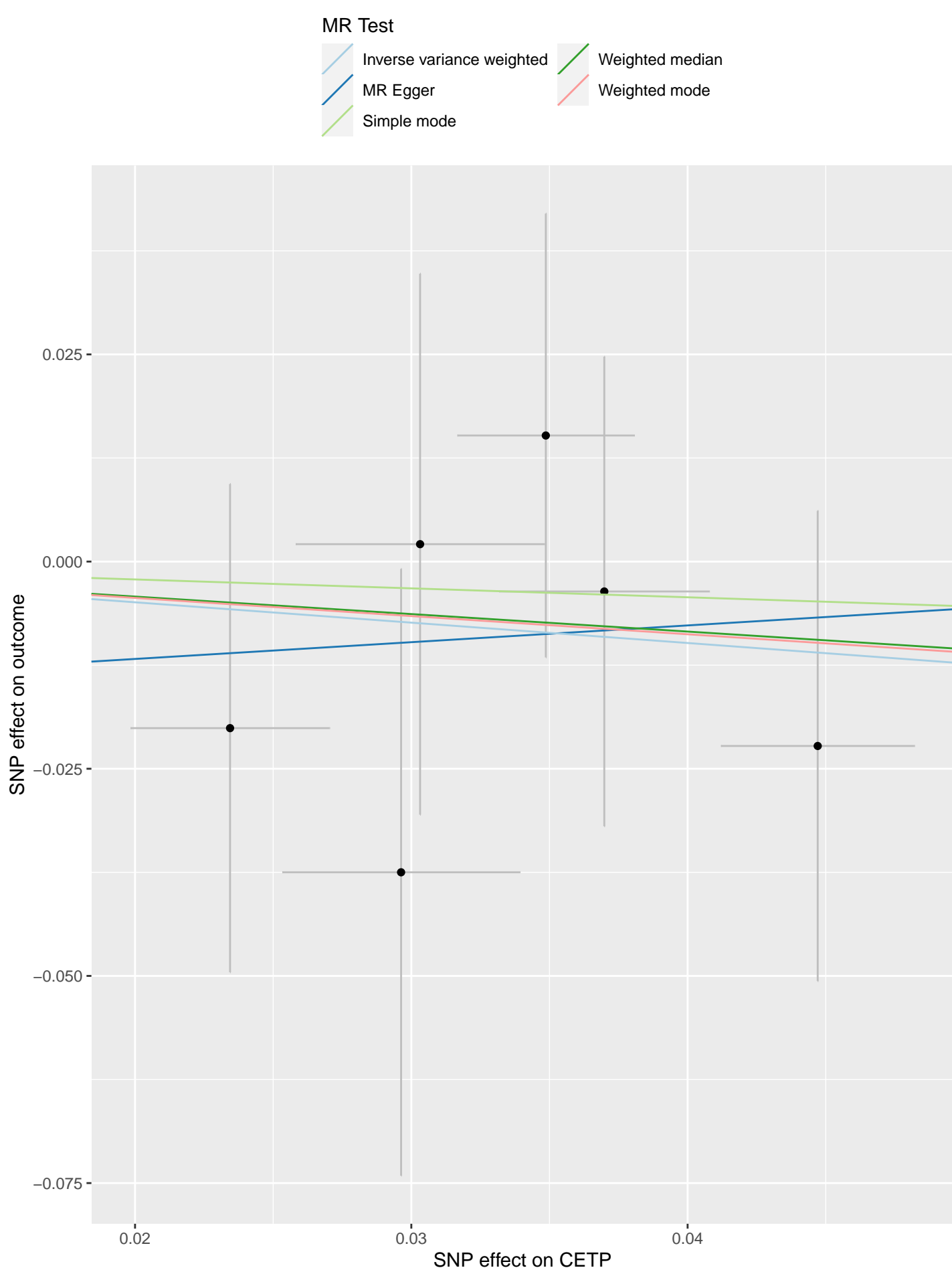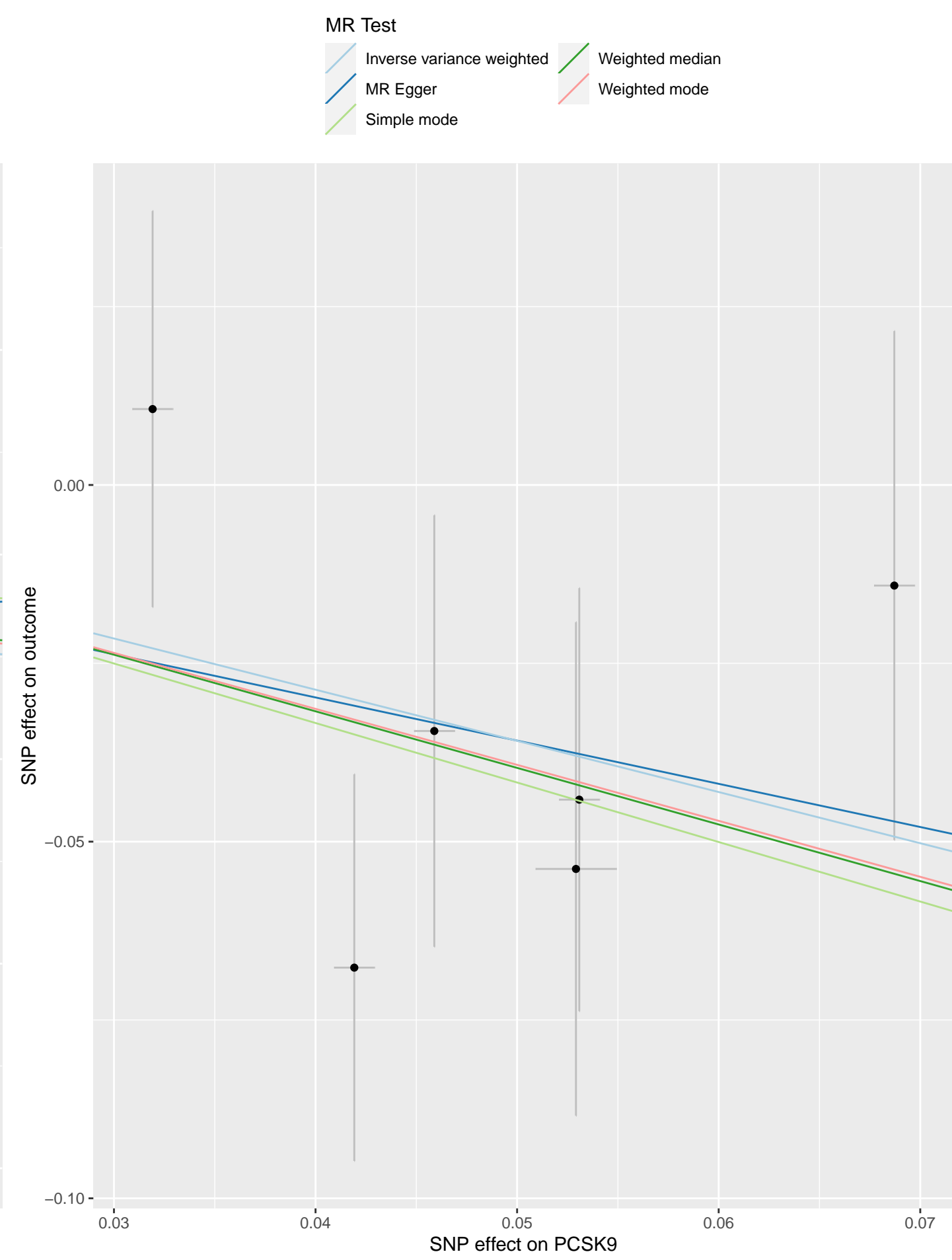

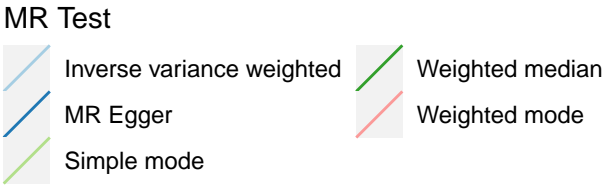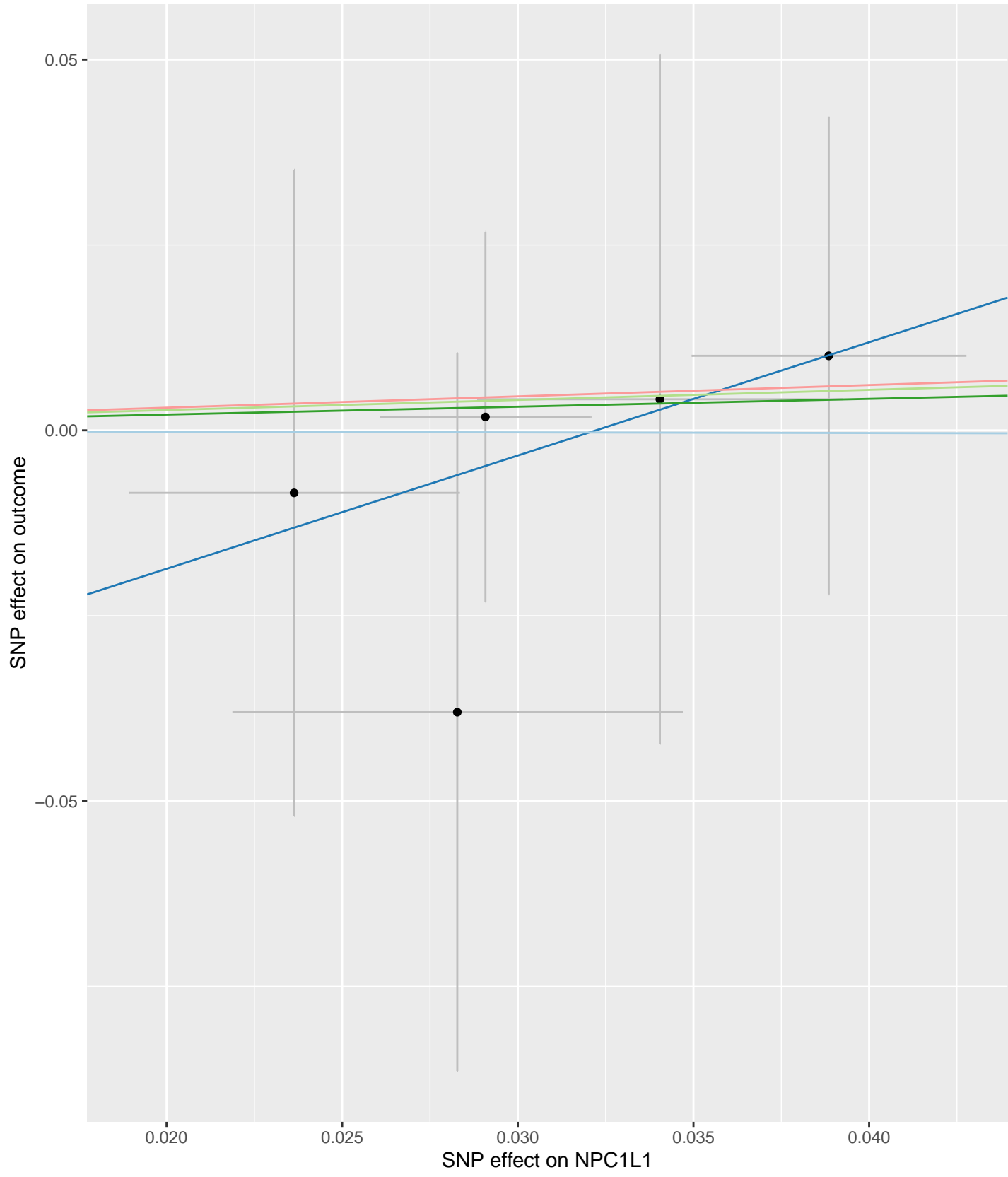

Supplement: S3 Fig — (PDF) [file pgen.1009525.s017.pdf]

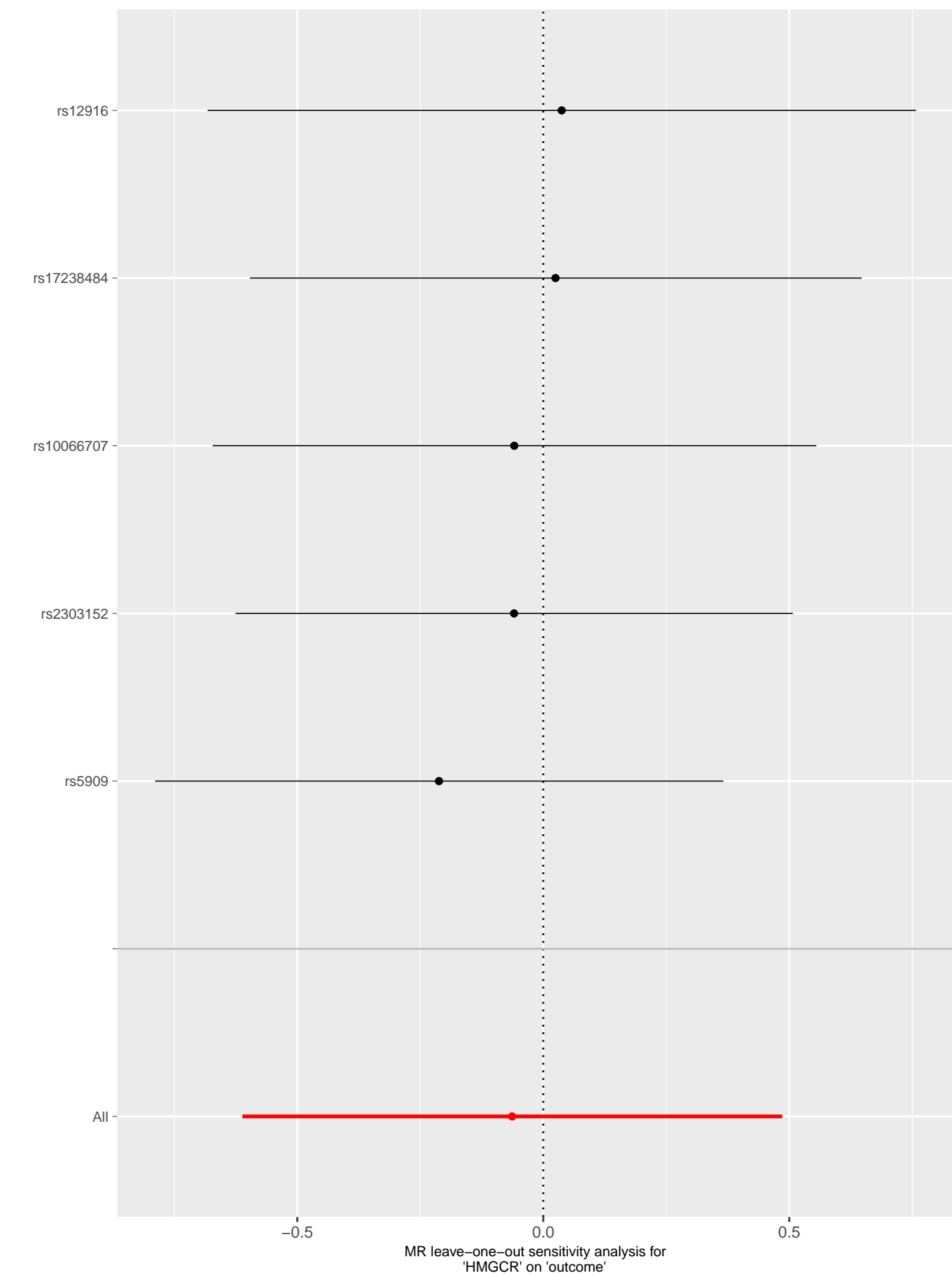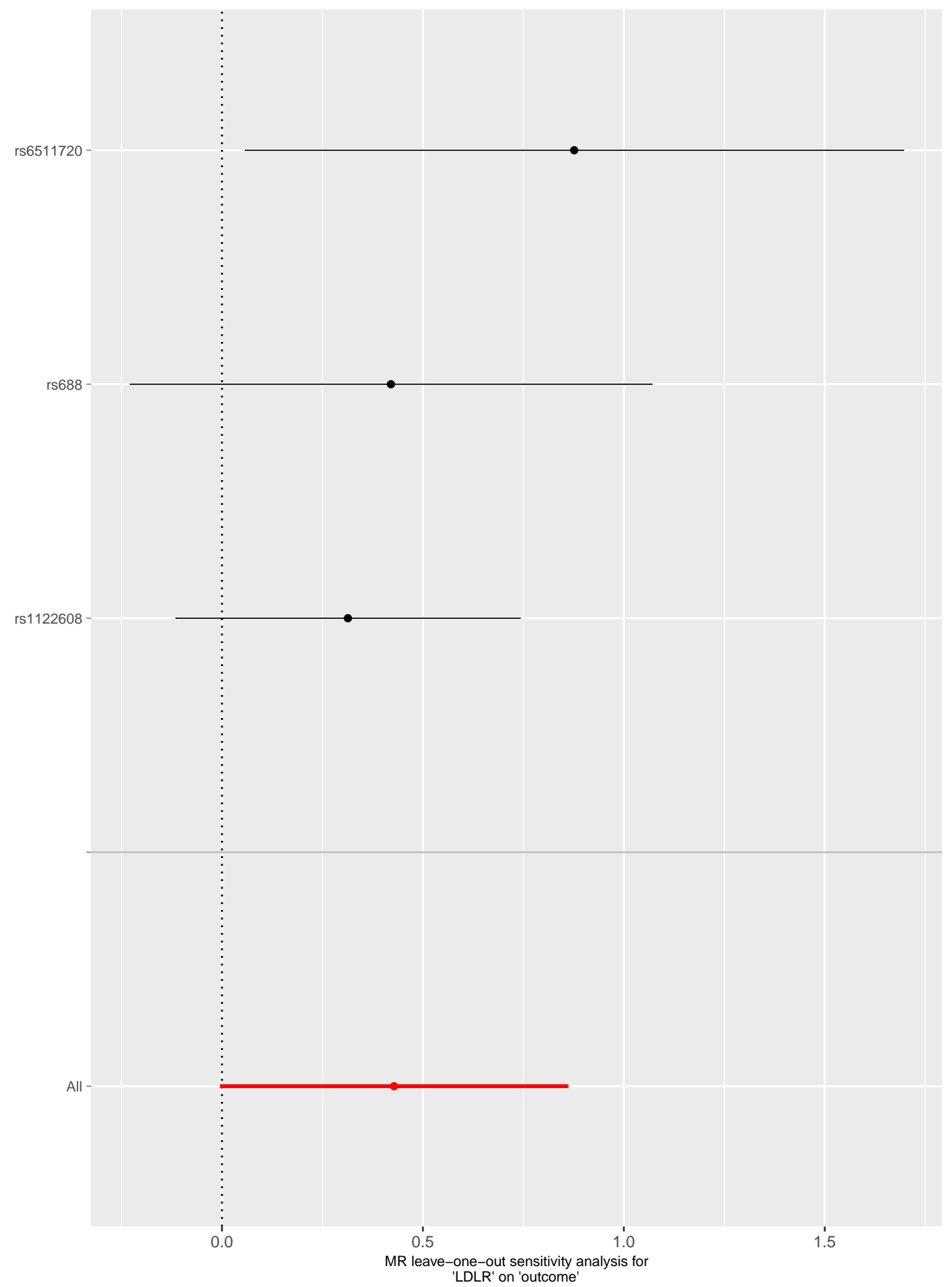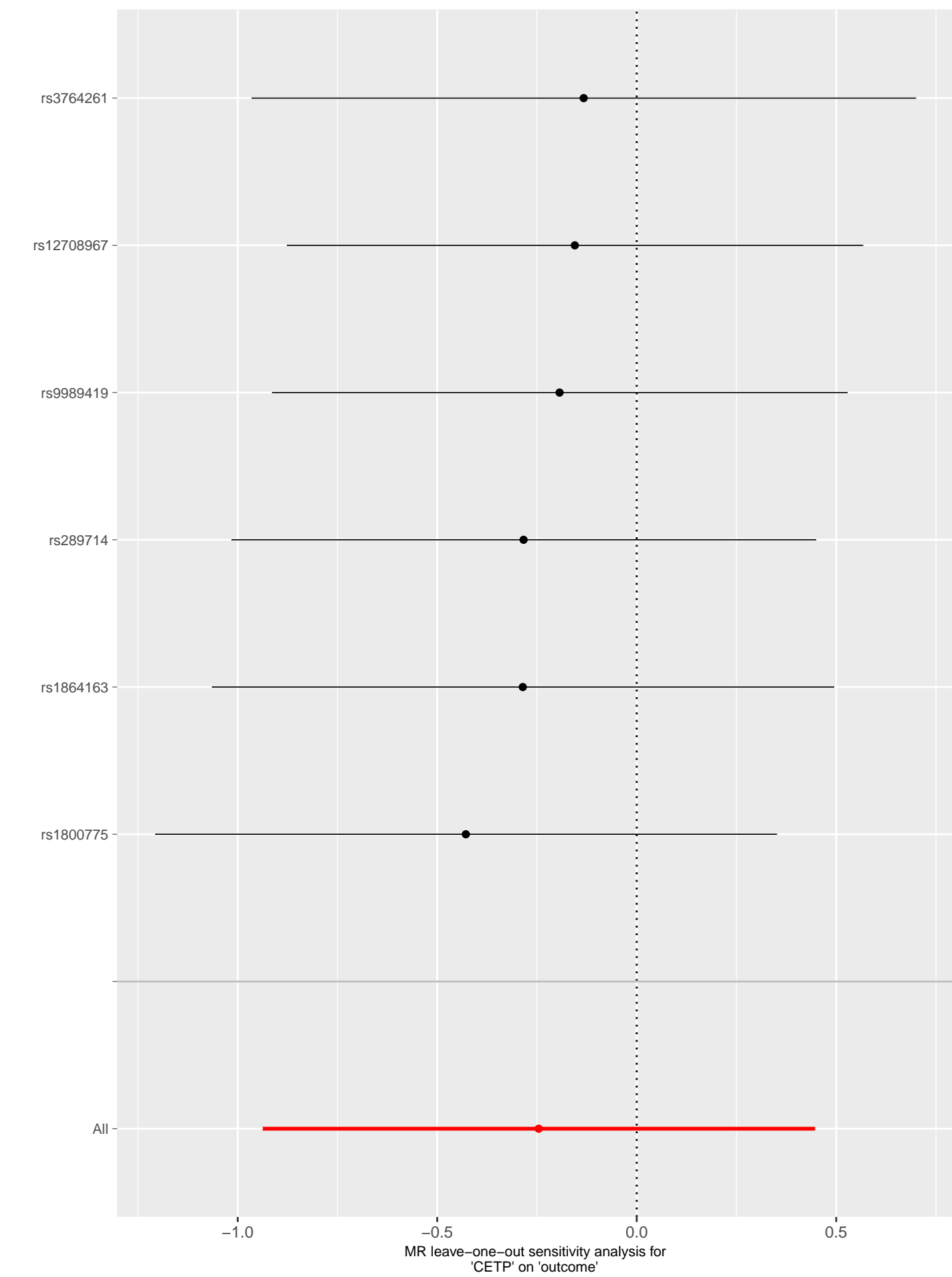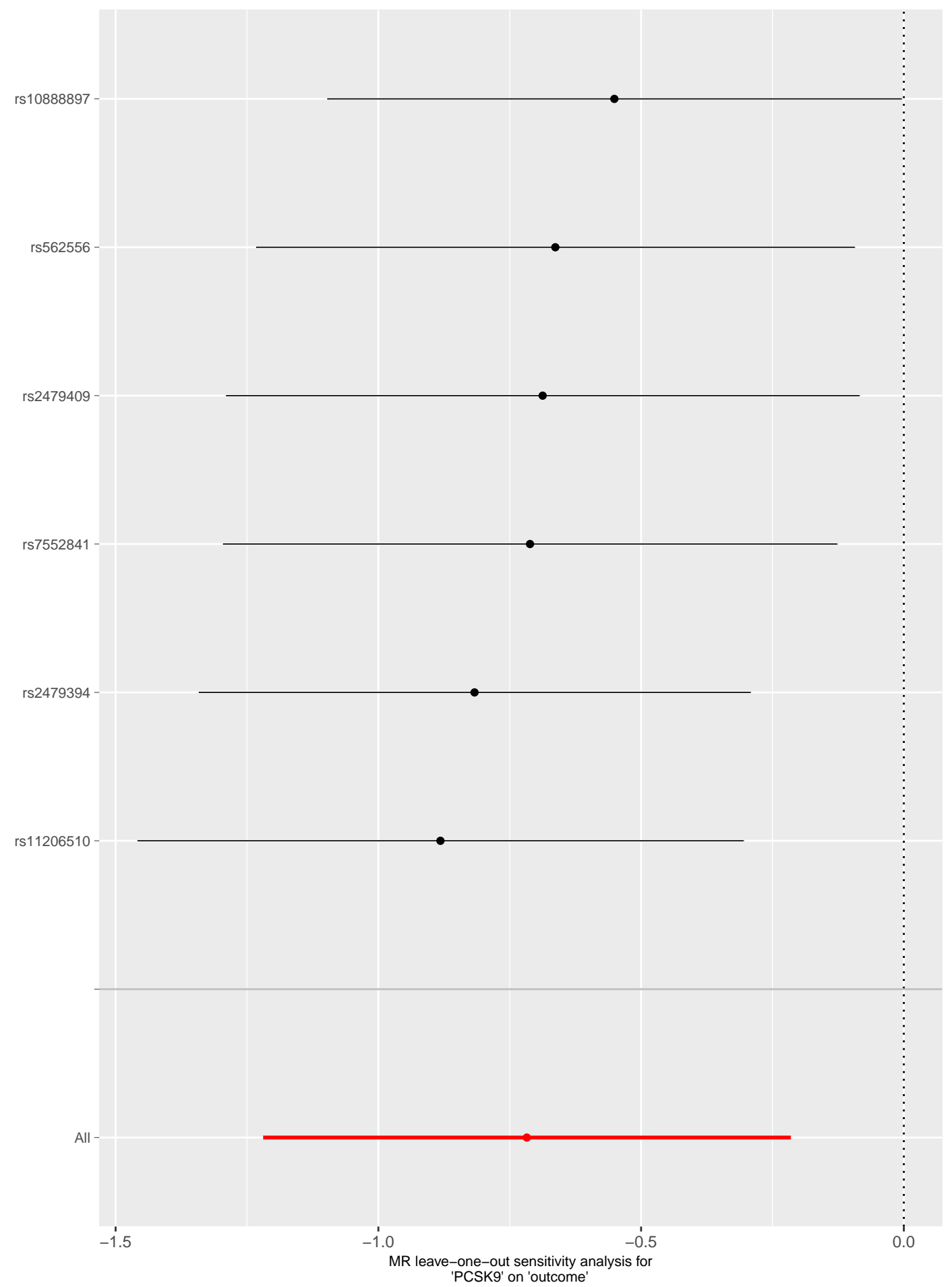

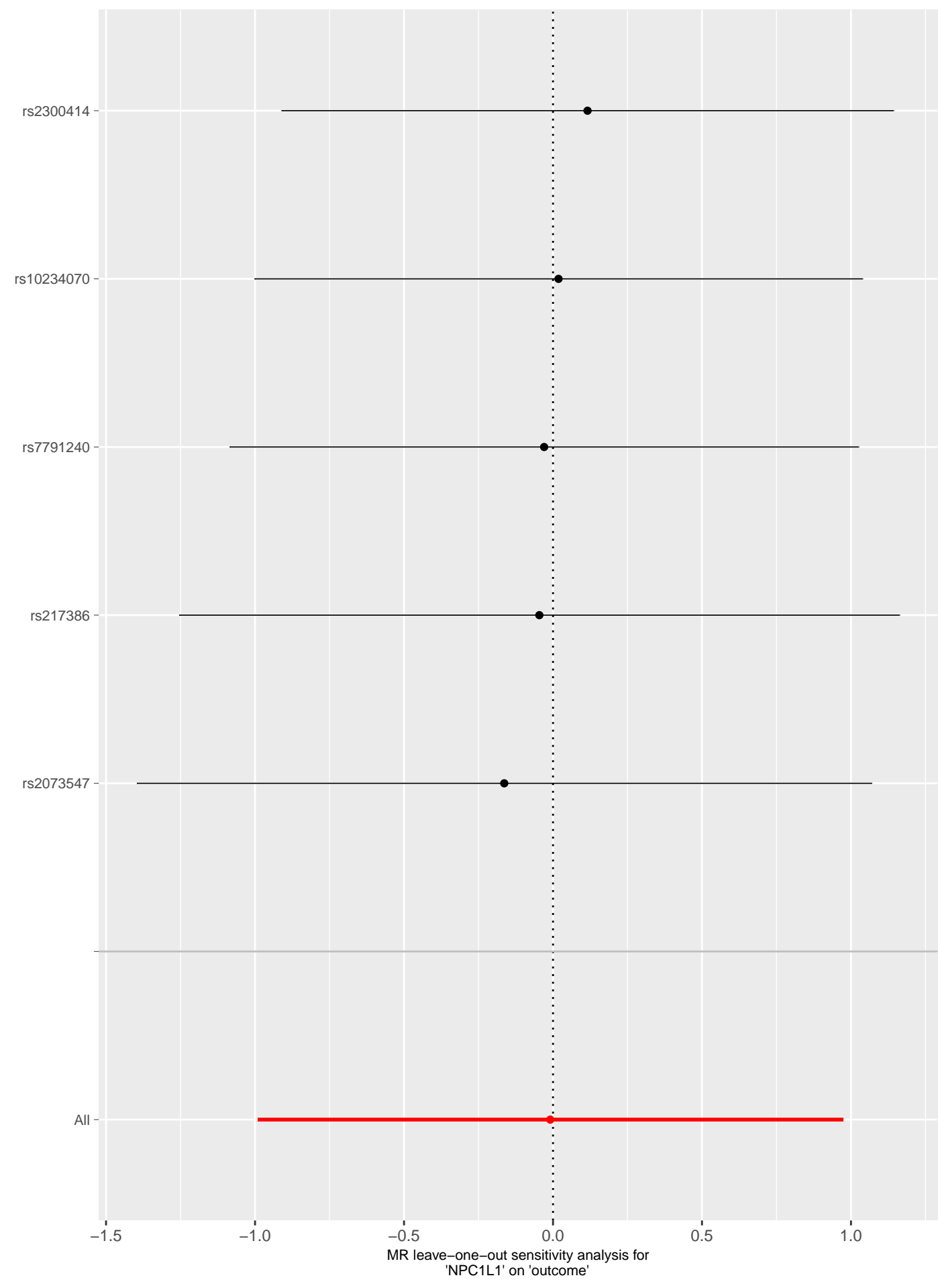

Supplement: S4 Fig — (PDF) [file pgen.1009525.s018.pdf]

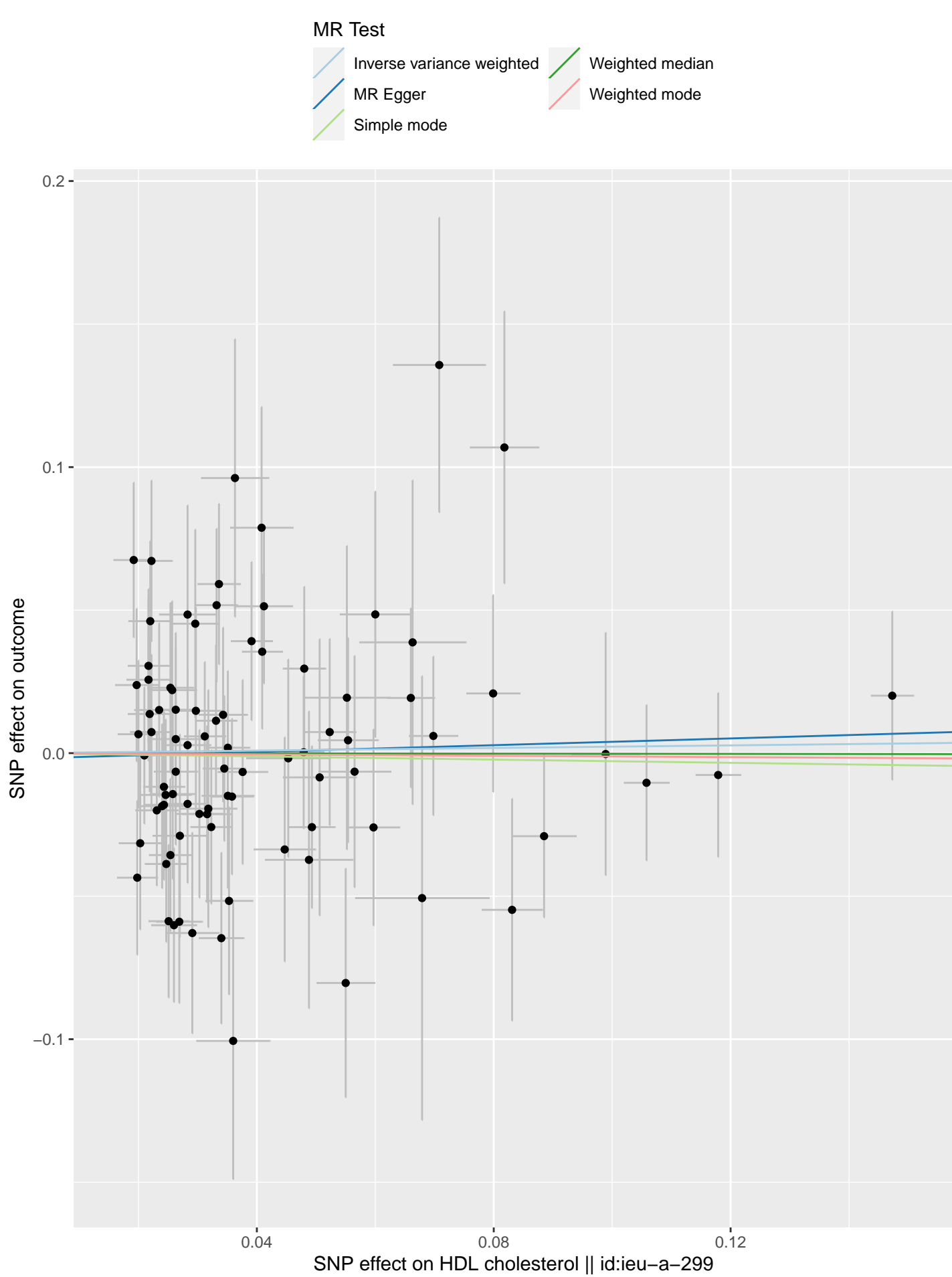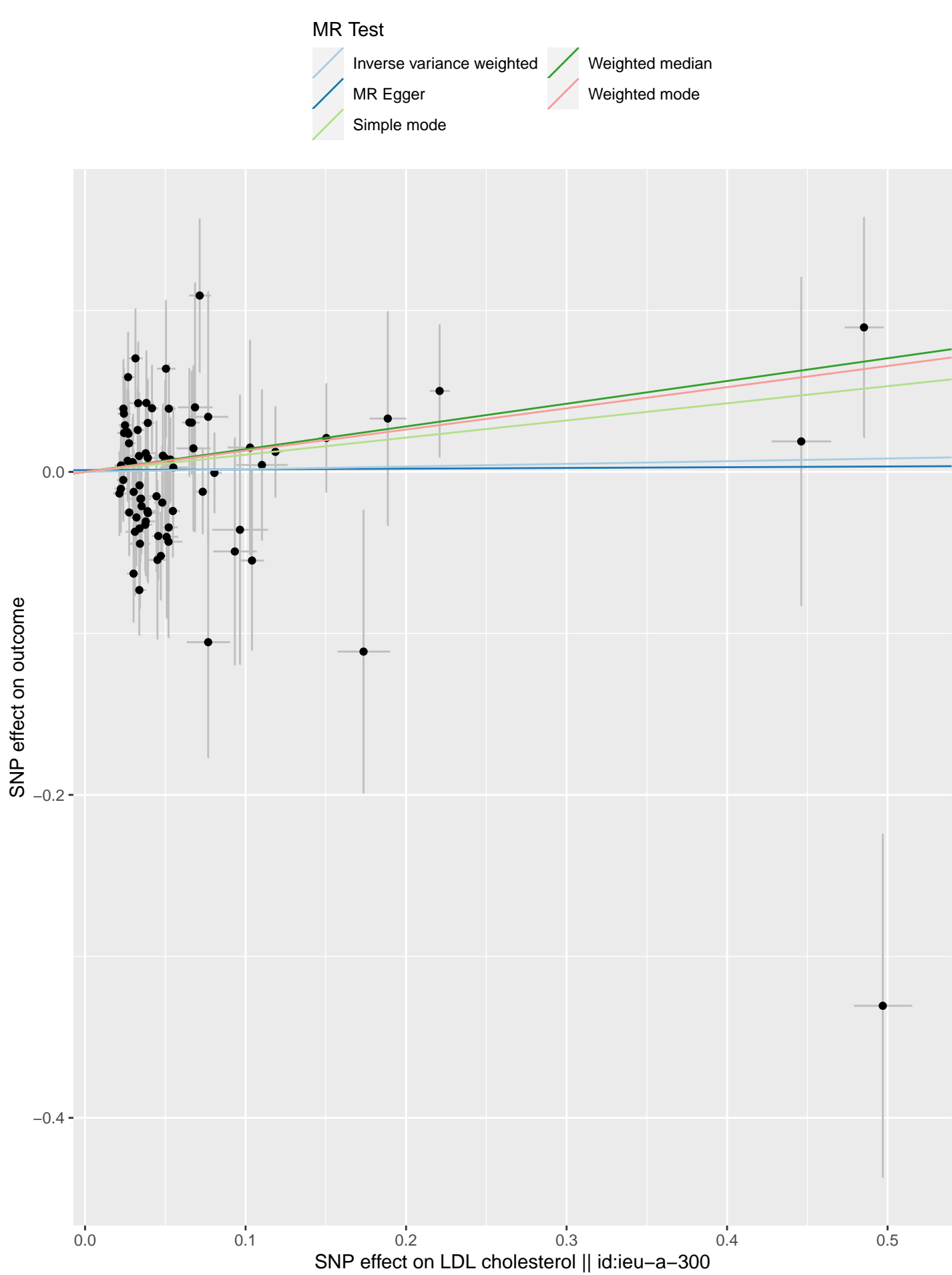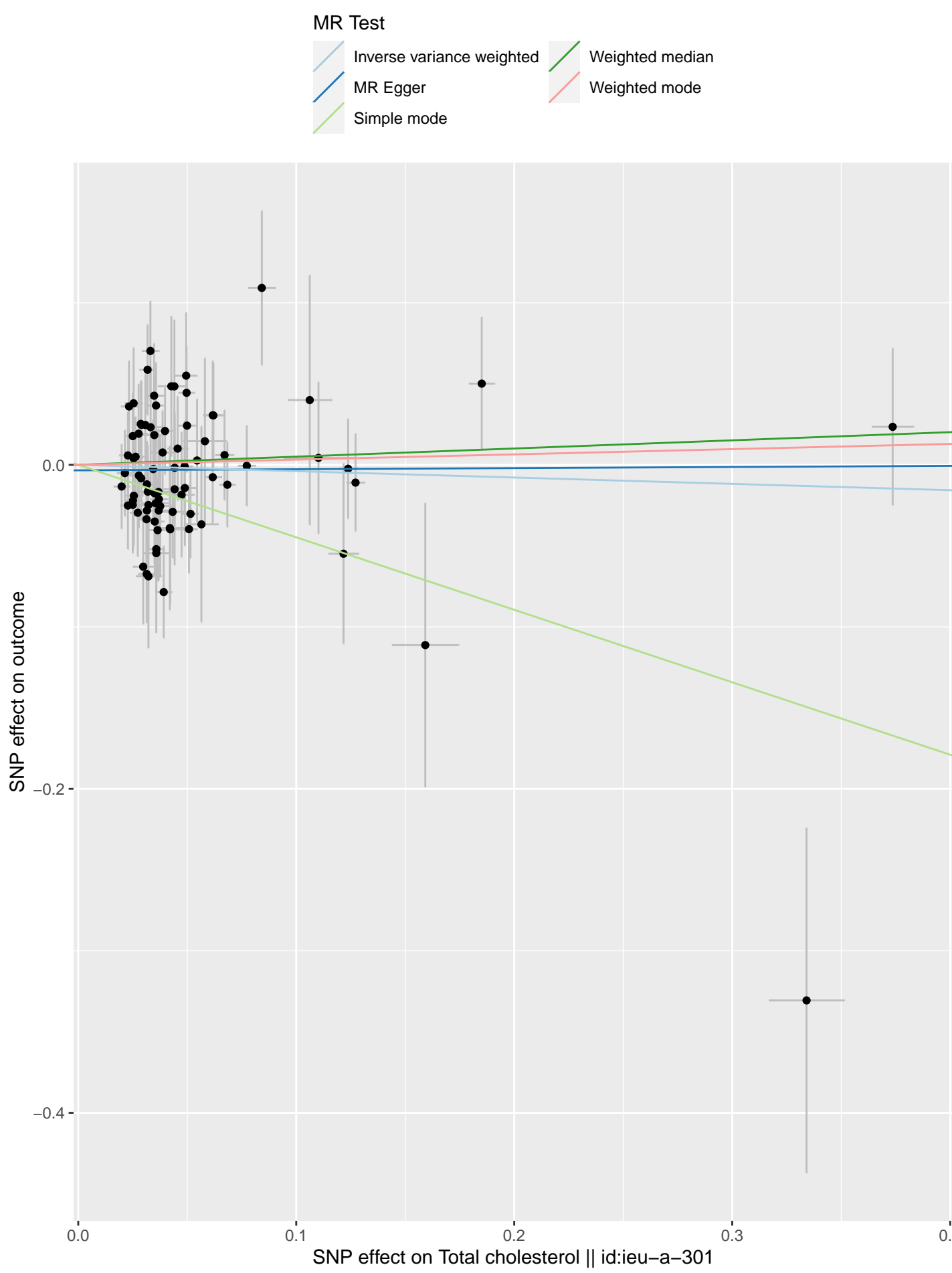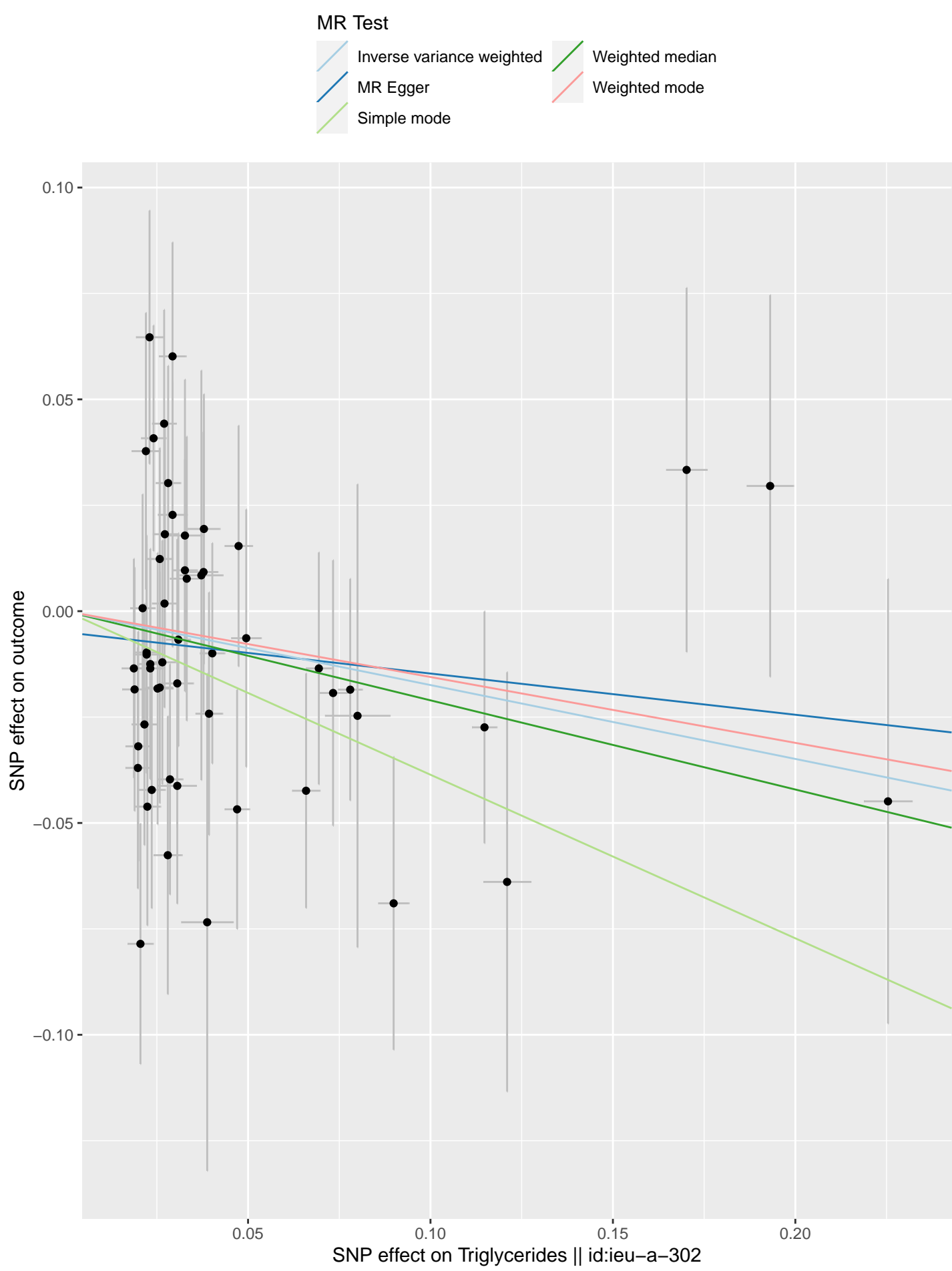

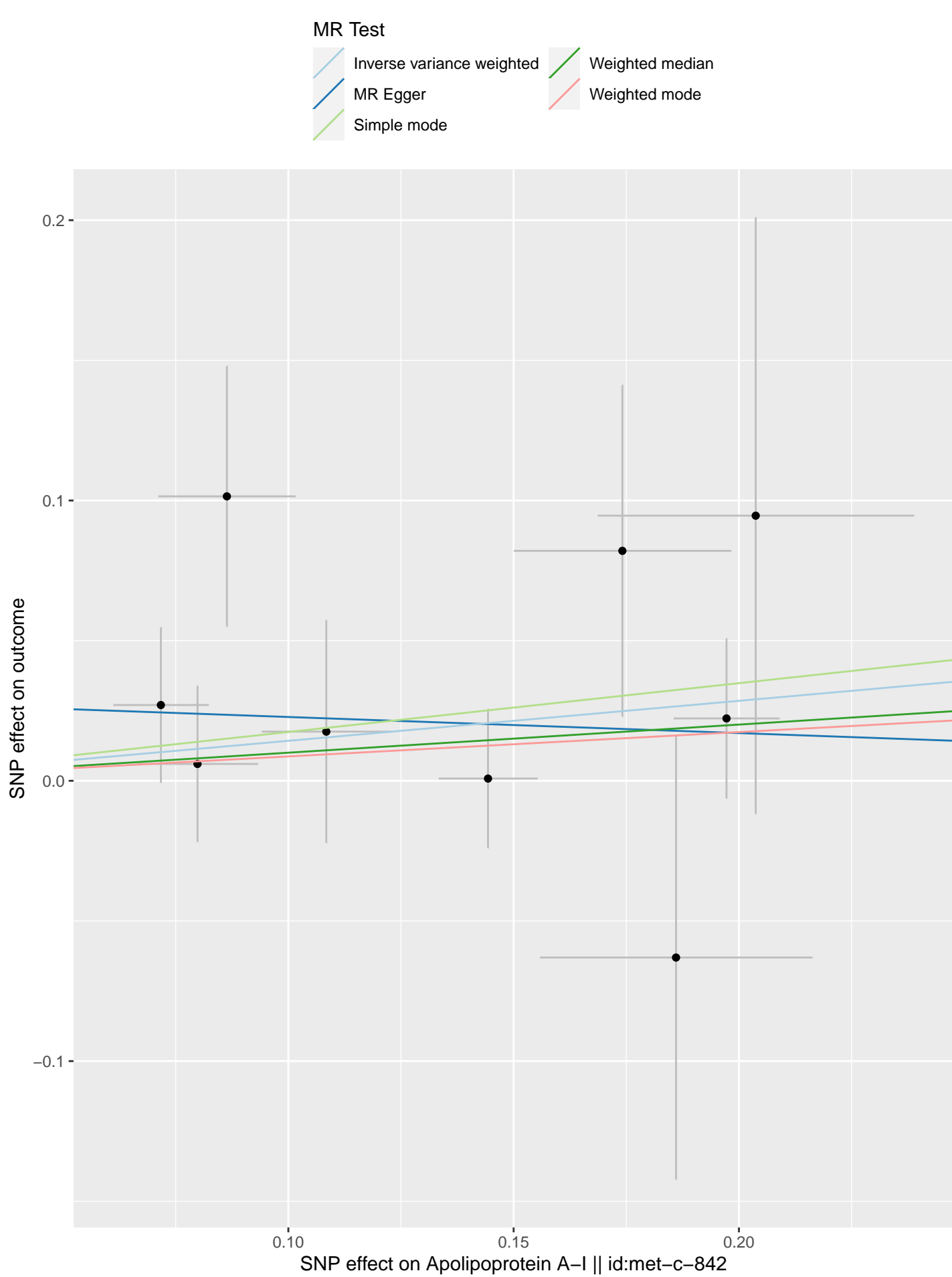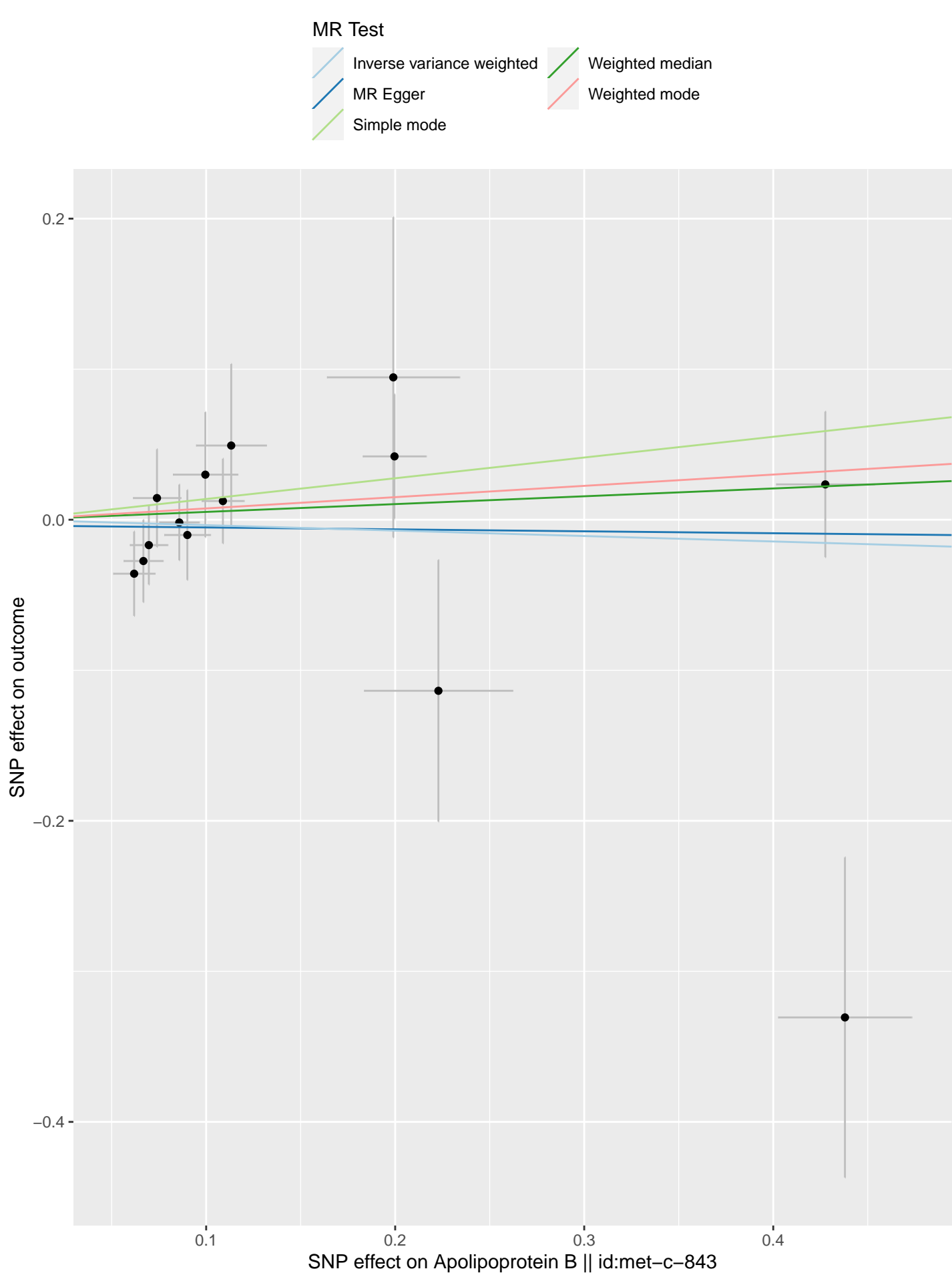

Supplement: S5 Fig — (PDF) [file pgen.1009525.s019.pdf]

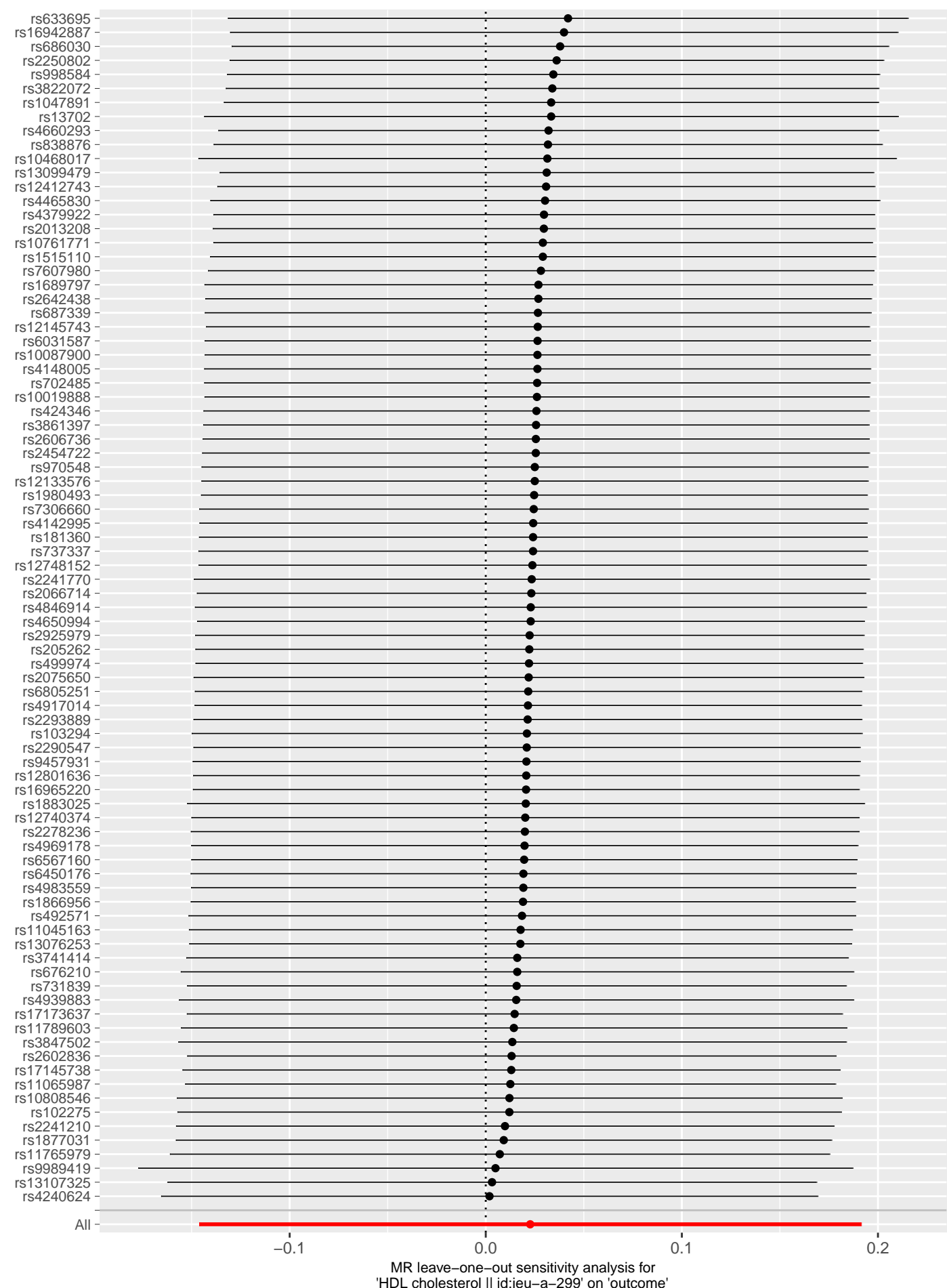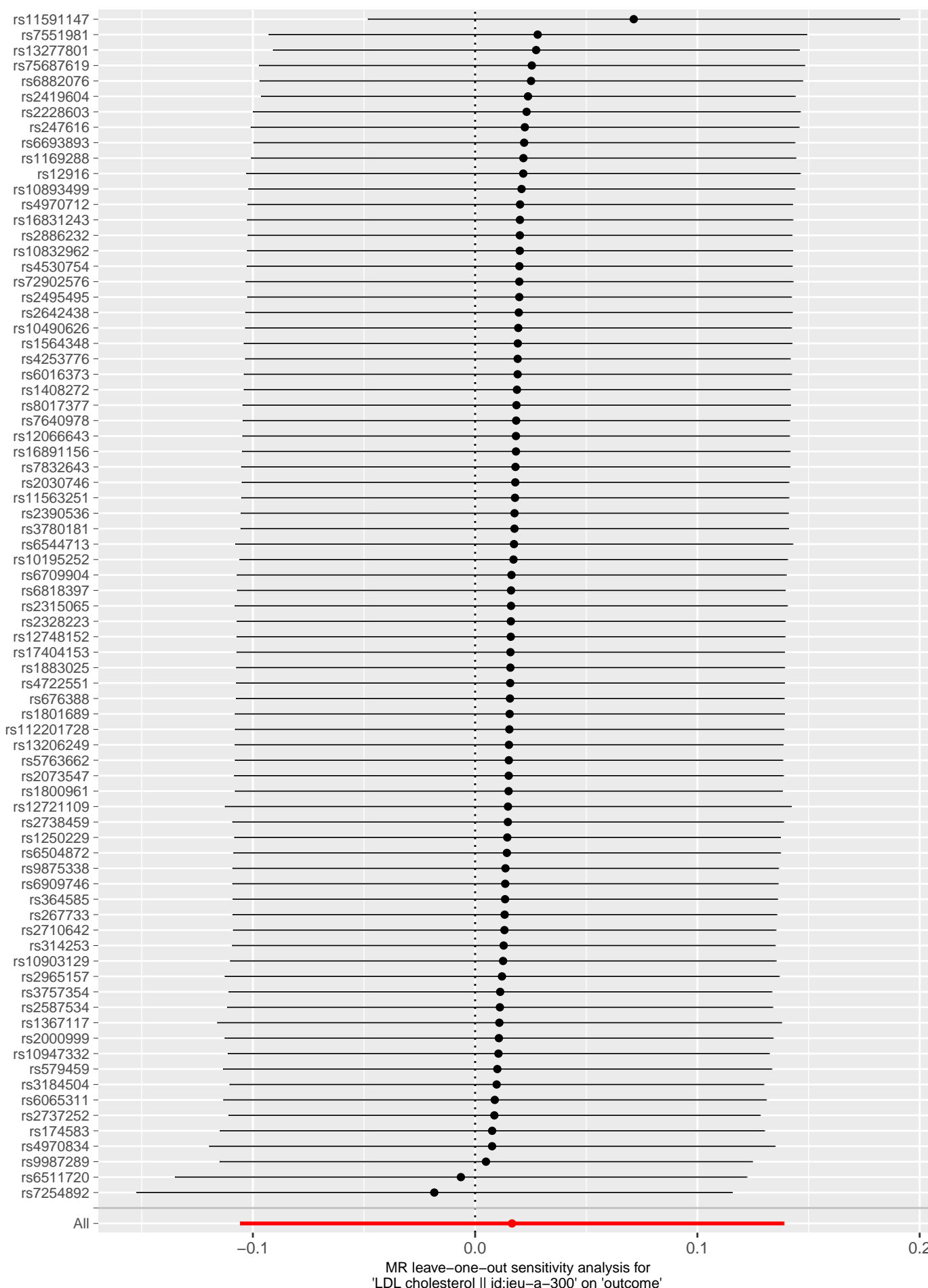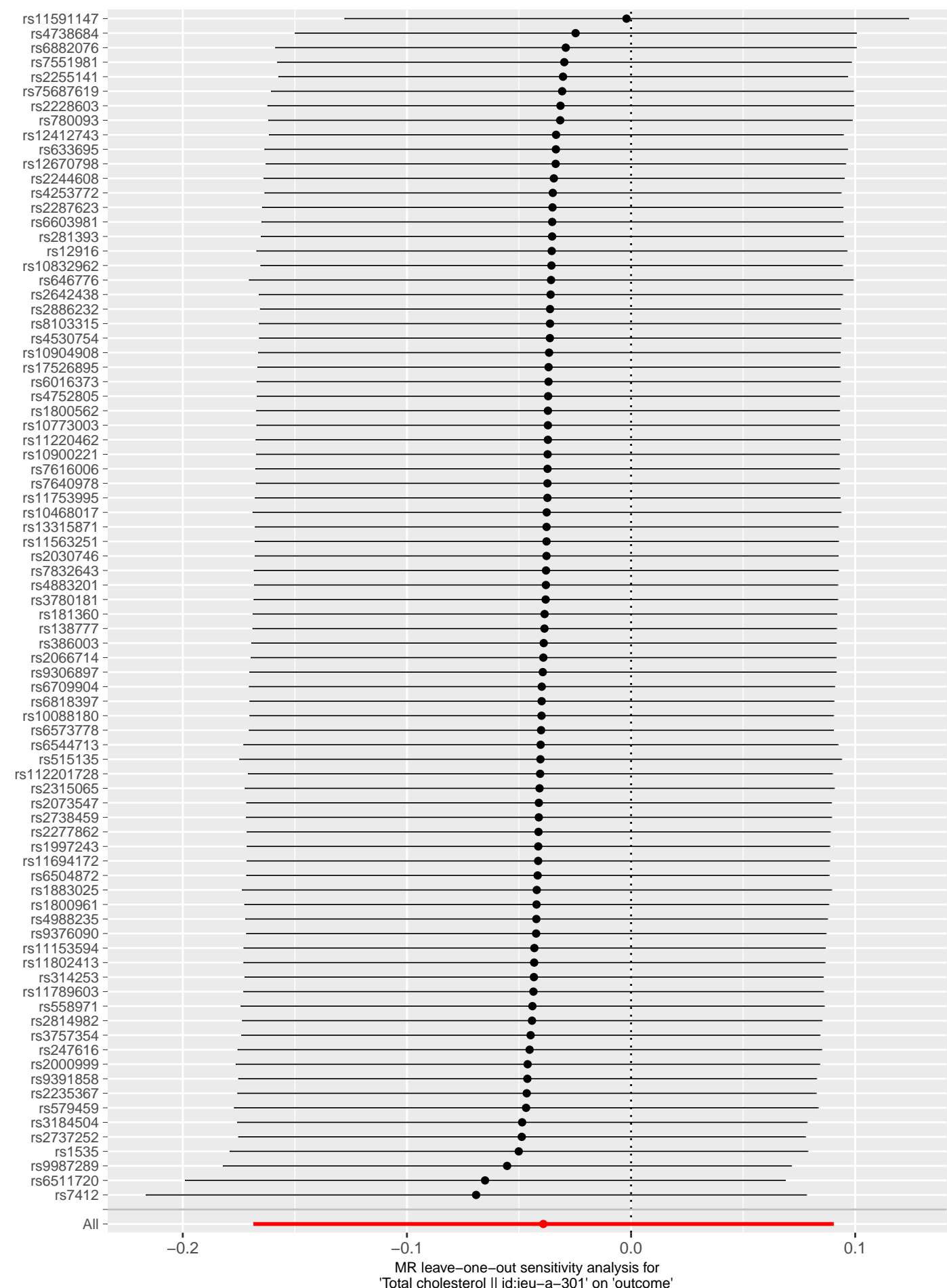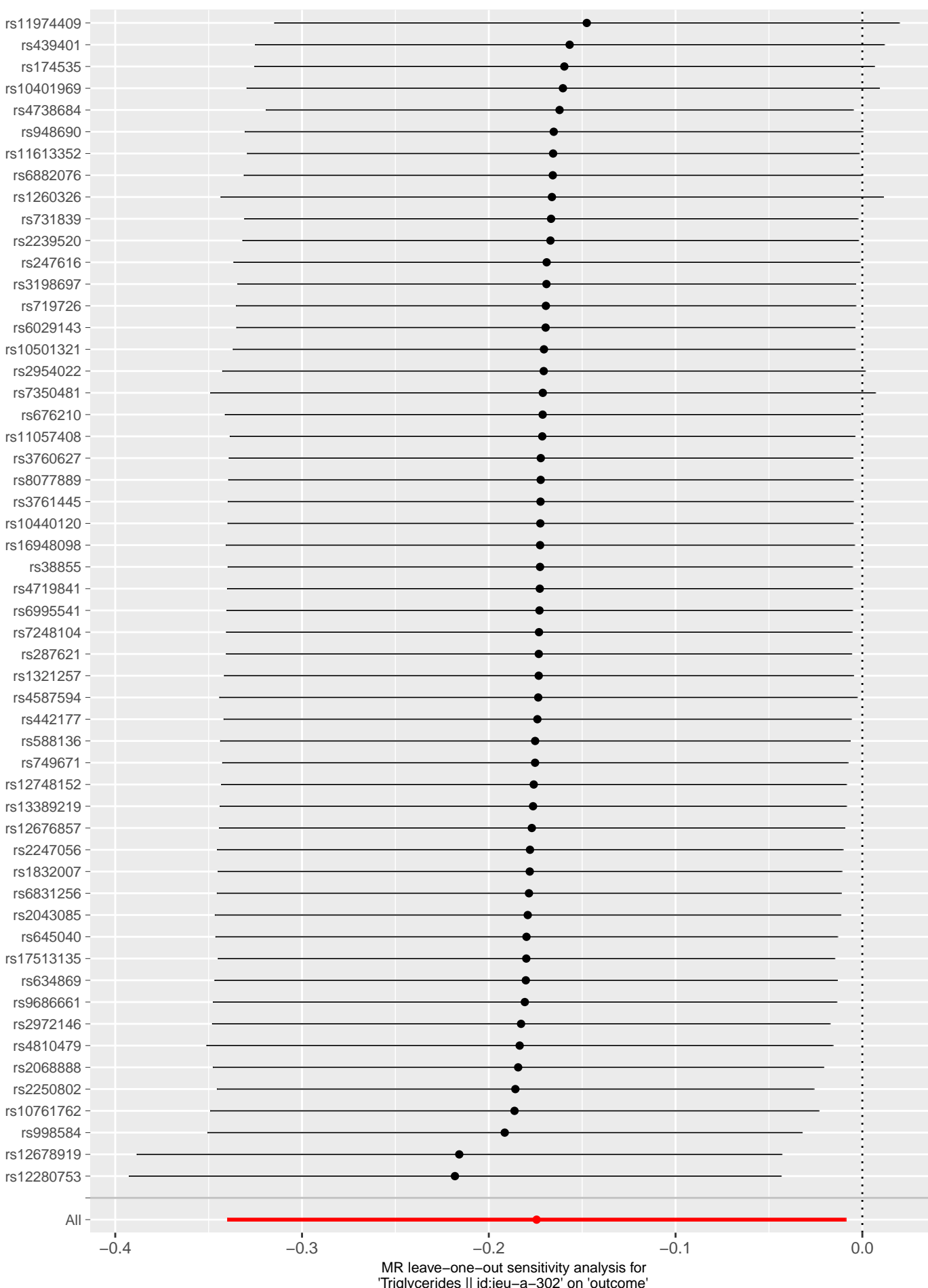

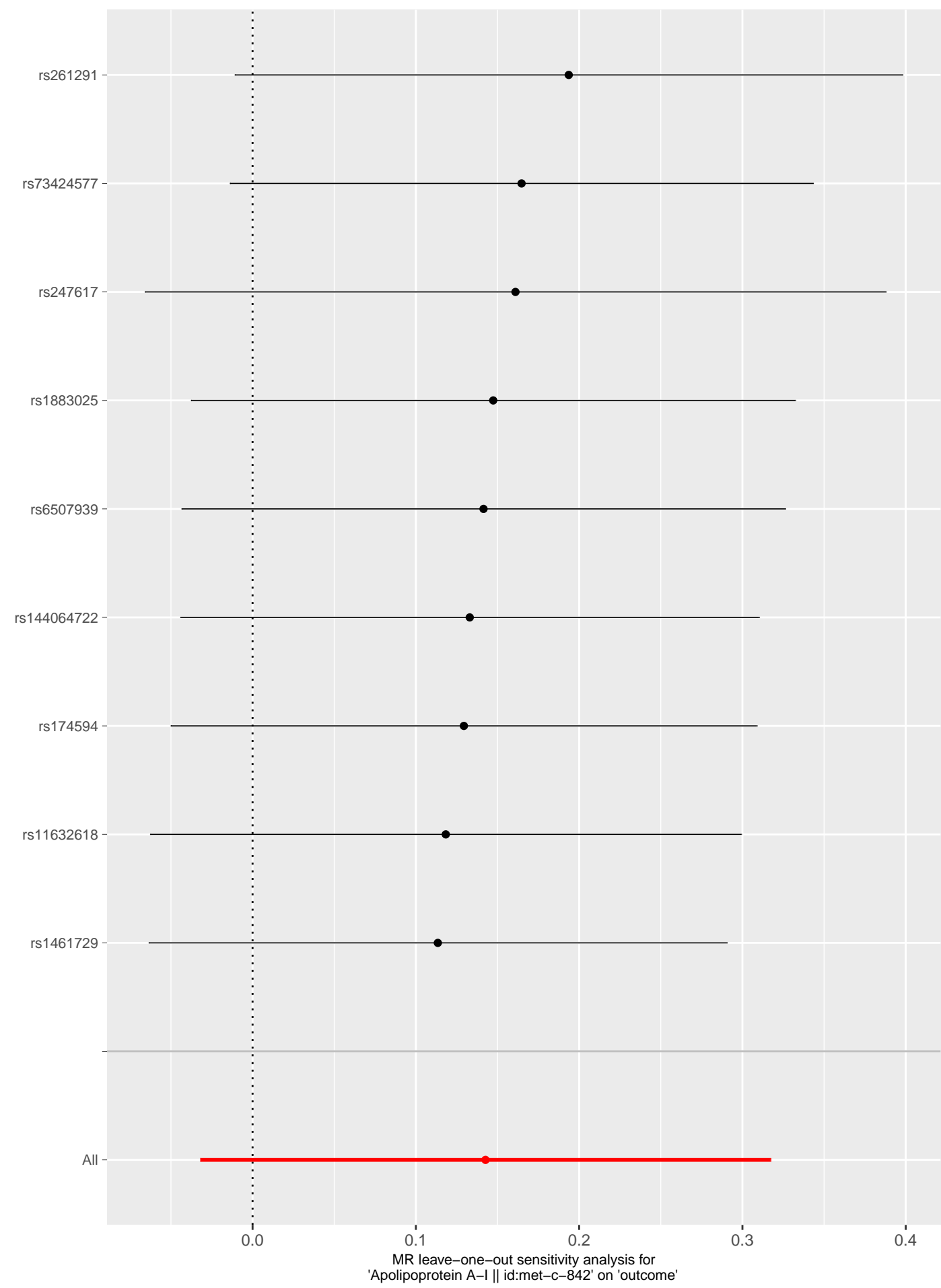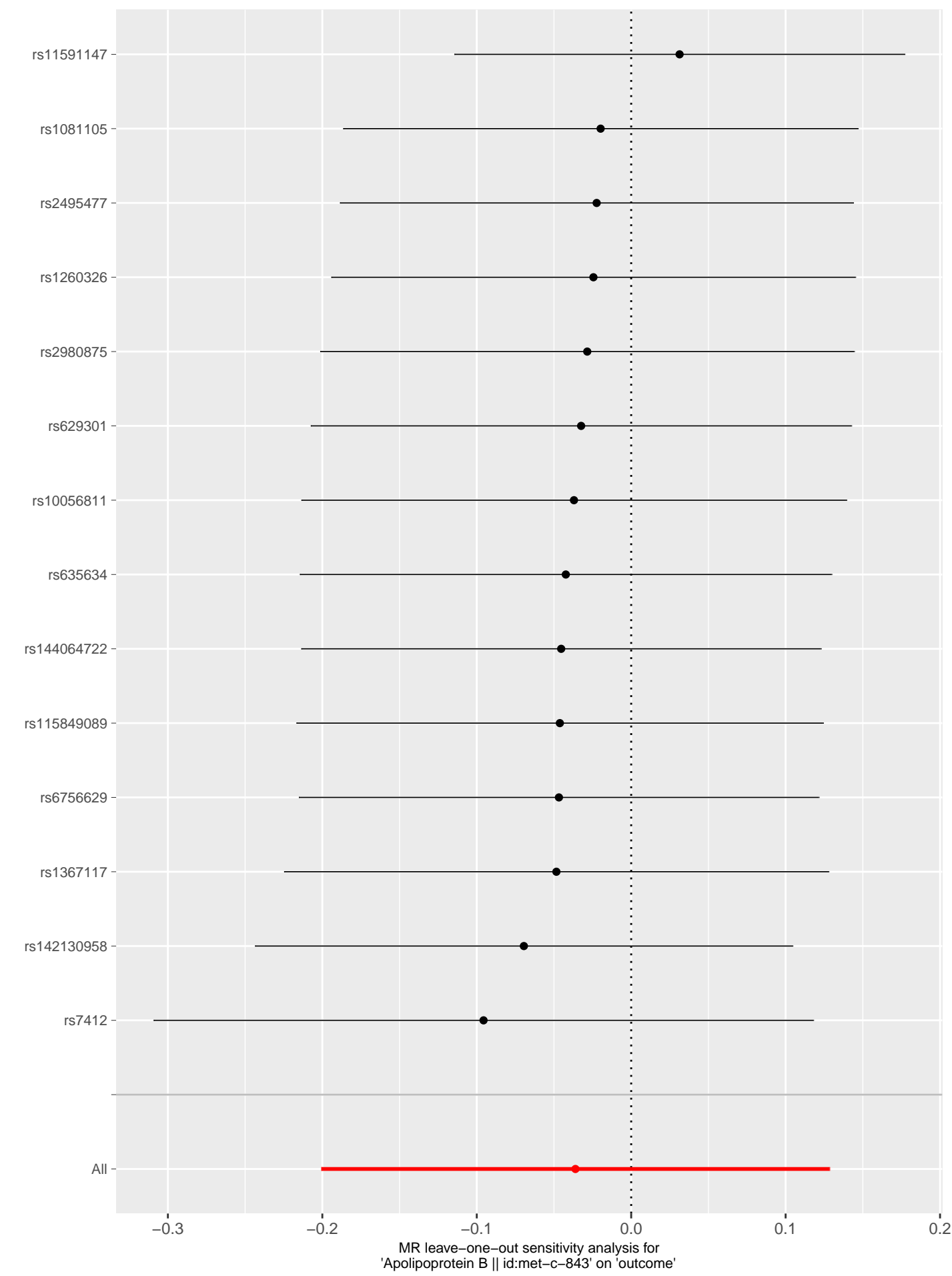

Supplement: S6 Fig — (PDF) [file pgen.1009525.s020.pdf]

**HMGCR**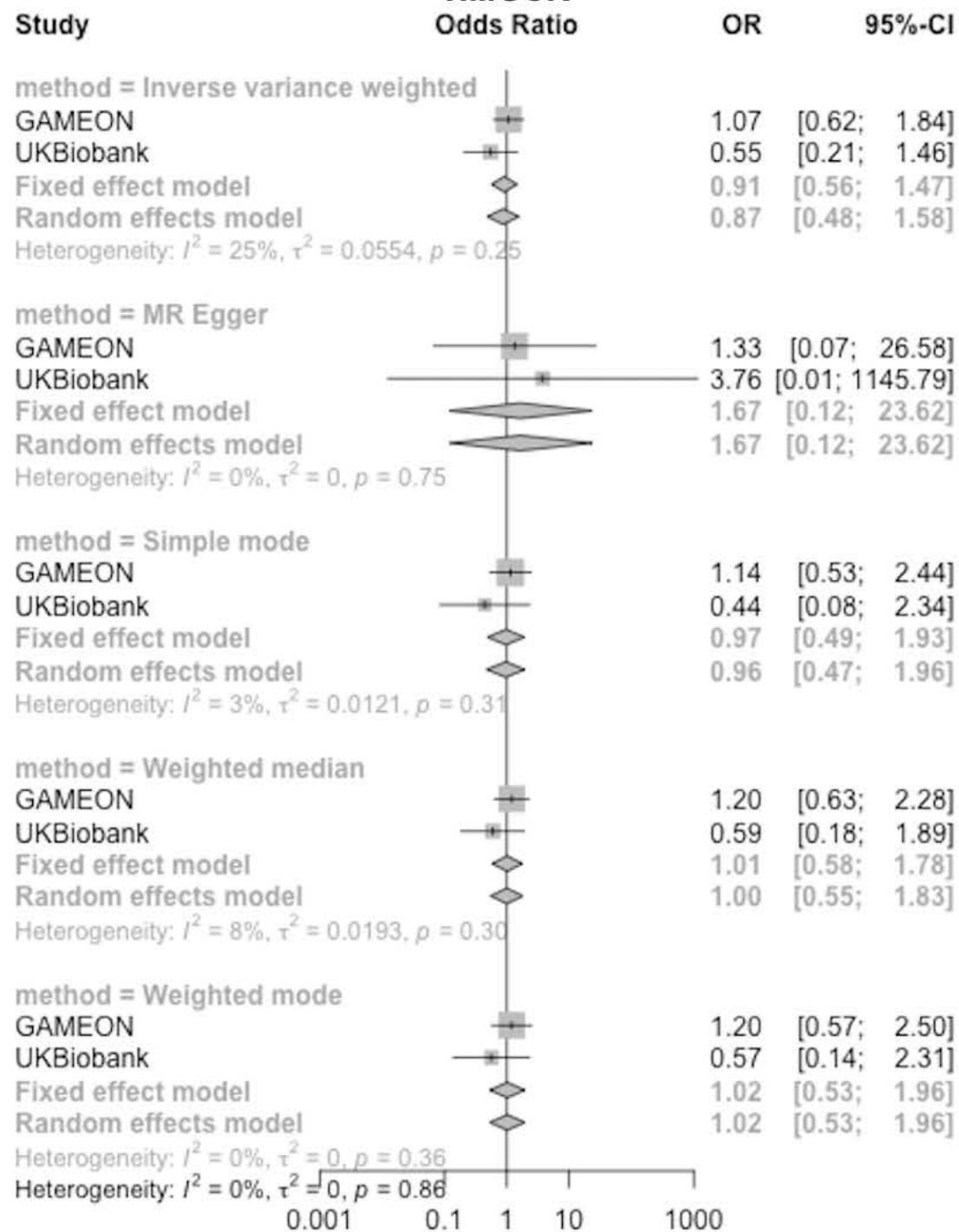**NPC1L1**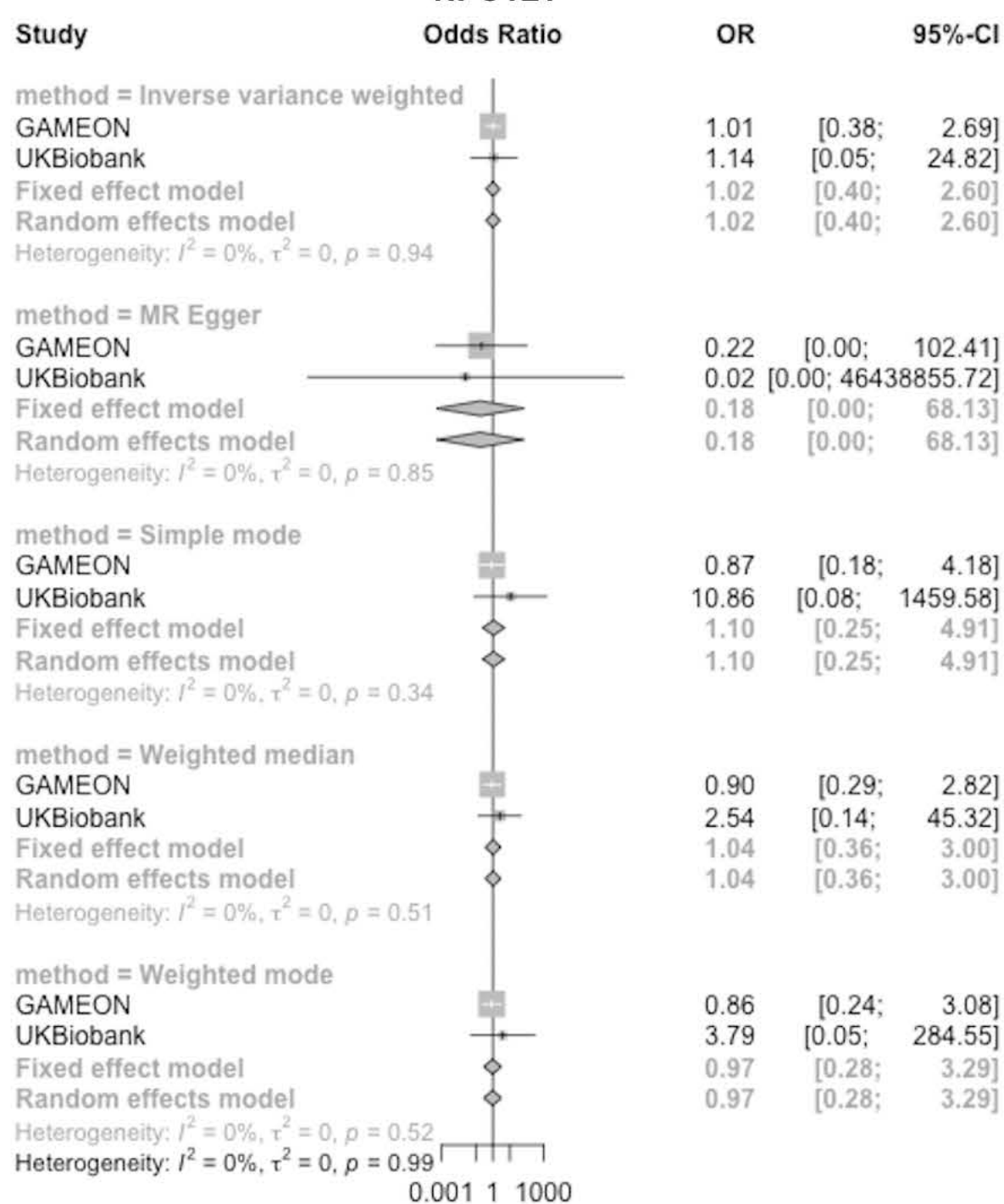

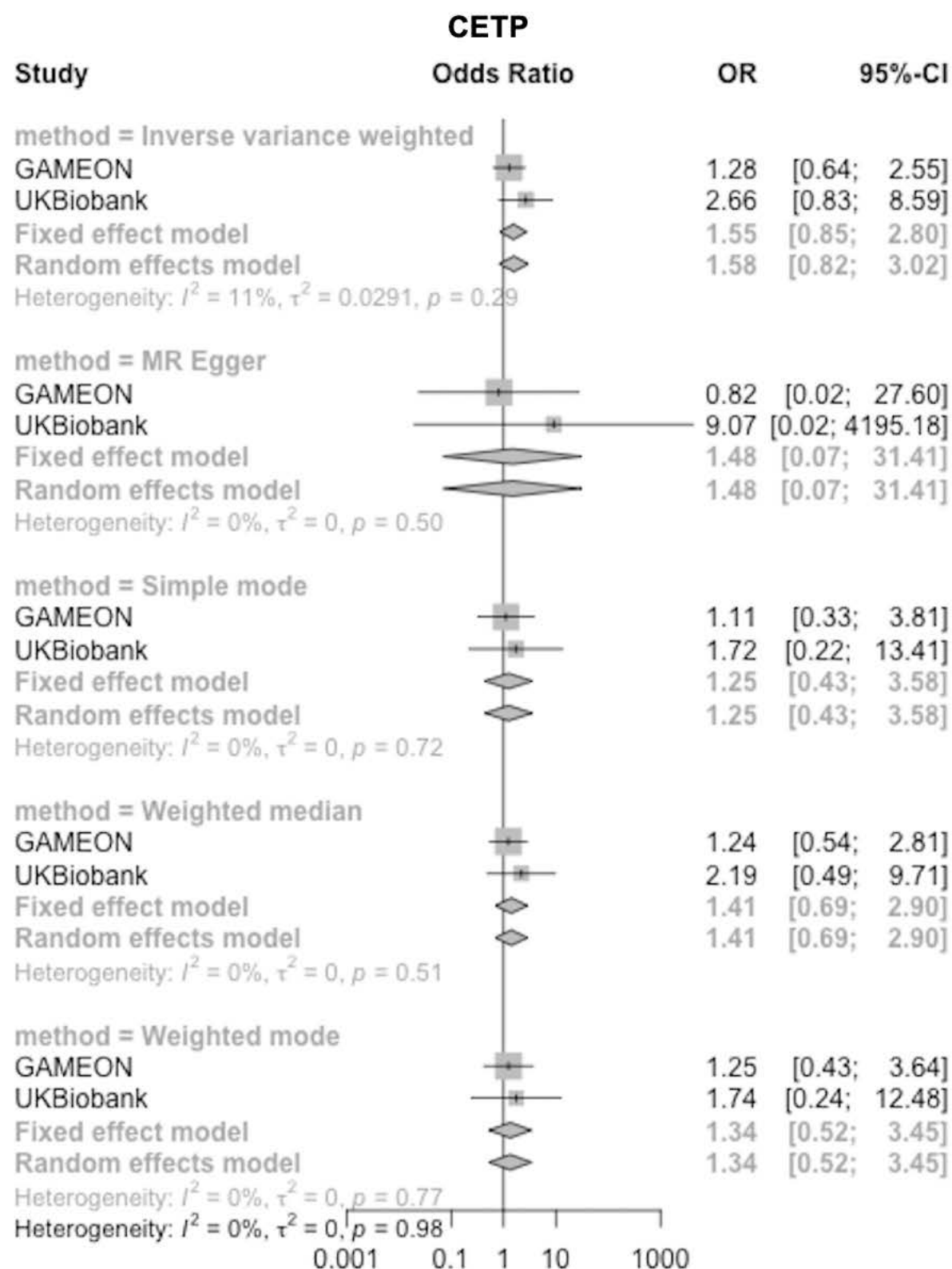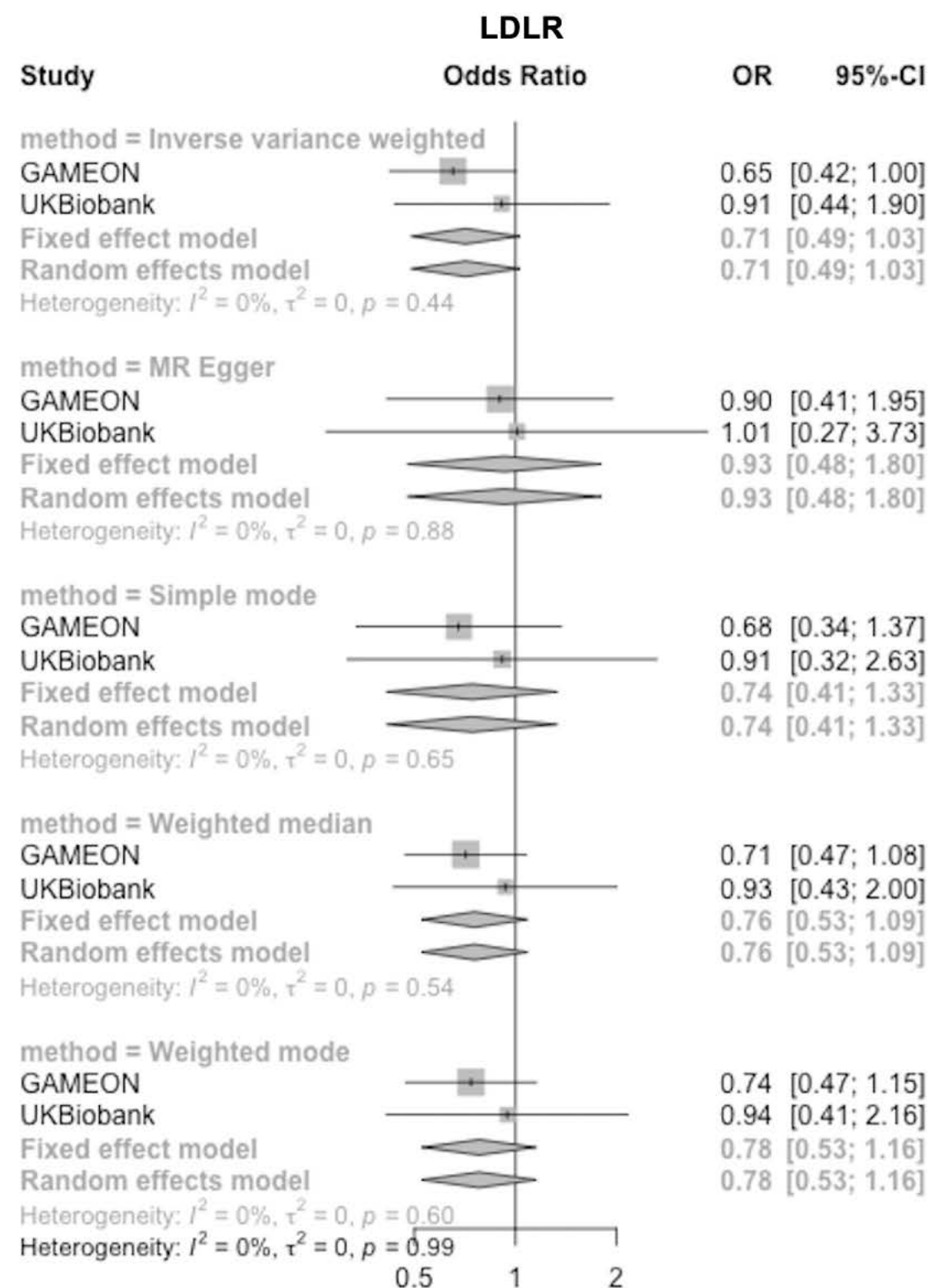

# PCSK9

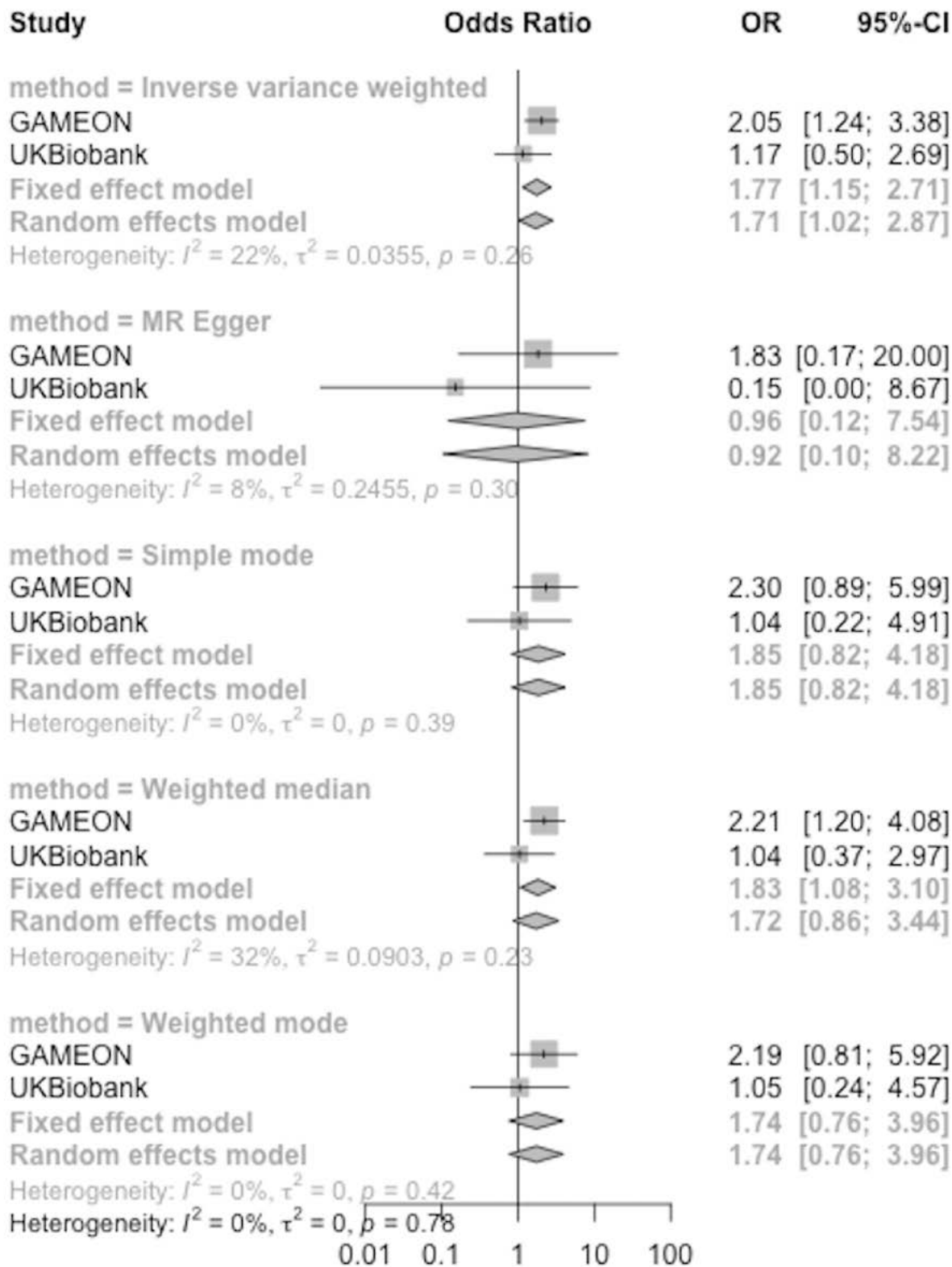

Supplement: S7 Fig — (PDF) [file pgen.1009525.s021.pdf]
