# Supplementary material for: Using genetic variants to evaluate the causal effect of cholesterol lowering on head and neck cancer risk: A Mendelian randomization study
Source: PLoS Genet. 2021 Apr 22;17(4):e1009525. doi: 10.1371/journal.pgen.1009525 (PMC8096036; doi:10.1371/journal.pgen.1009525)
Supplement: Attachment — Submitted filename: Response_to_reviewers_final.docx [file pgen.1009525.s022.docx]

**Response to referees**

Please find enclosed, a revised version of our paper entitled ‘Using genetic variants to evaluate the causal effect of cholesterol lowering on head and neck cancer risk: a Mendelian randomization study’.

We thank both reviewers for their comments and have responded to all points in order below. All revisions have also been highlighted in yellow within the revised manuscript.

**Reviewer #1:**

**This is very well written and explained manuscript. The study is well designed, with the relationship between cholesterol and head and neck cancer comprehensively studied. The analyses are appropriate, and the conclusions drawn sound. I only have minor comments for consideration.

Introduction: Were the observational studies assessing head and neck cancer risk and survival performed stratifying by OC and OPC? Given the differences in etiology for these cancers (e.g. HPV exposure) some discussion of this could be worthwhile.**

In response to this point, we have now added the following text to our Discussion:

*“A recent case-control study of over 11,000 participants found an inverse association between statin use and the occurrence of HNSCC (OR 0.86, 95%CI 0.77, 0.95, p= <0.01, of prior statin exposure for cases compared to propensity score‐matched controls) [1]. However, this observational study failed to stratify by anatomical subsite, which may have revealed distinct associations given differences in aetiology (e.g., the strong association of HPV infection with OPC).” (lines 591-596)*  **While I appreciate that the authors haven’t overstated their findings, some mention of multiple testing should be made in the manuscript.**

We take on board the reviewer’s concerns about multiple testing and have added the following to our manuscript:

*The combined OC/OPC results were robust to multiple testing in the main analysis conducted (i.e., IVW for the drug targets = p <0.05/5 = 0.001). (lines 401-402)*

**Reviewer #2:**

**This is an interesting manuscript investigating the association of cholesterol lowering in relation to head and neck cancer risk using Mendelian randomization. The research is well-conducted. I have a few comments/suggestions which may further improve this work.

1) Given that head and neck cancer is a relatively rare cancer in the western world and that the number of cases was 6K in the current study, I would have liked to see a power analysis to check the minimum detectable OR and compare to findings observed in observational studies.**

We have now carried out power calculations as suggested and included detail on these below, however many argue that post-hoc power calculations are conceptually flawed and can also be analytically misleading [2]. They do not indicate true power for detecting statistical significance, as observed power differs from the true power and the true effect size remains typically unknown. Furthermore, observed (or post-hoc) power and *p*-values are directly related and so reporting post-hoc power is nothing more than reporting the *p*-value in a different way [2]. Therefore, we would prefer not to include these in our revised manuscript, however if you or the editor feel strongly, the following text and supplementary figures could be included:

*“Post-hoc power calculations were performed which showed there was 80% power to detect an odds risk of 1.6 or above (with a variance explained of 0.5%, which is the R^2^ value for the PCSK9 instrument), in the combined meta-analysis with GAME-ON and UK Biobank. We therefore had adequate power to detect the effect observed for the main PCSK9 finding, however this was not the case at a subsite level when stratifying by OC or OPC.”*

**Post-hoc power calculations for oral and oropharyngeal cases in both GAME-ON and UK Biobank.**

**2) The authors were very detailed in the sensitivity analyses they performed to probe into potential violations of MR assumptions. One analysis that was missing was a co-localisation analysis that can investigate whether the positive findings for PCSK9 and LDLR are due to violation of the exclusion restriction assumption due to LD.**

We have now completed colocalisation analysis for *PCSK9* and *LDLR*.

The following has been added to our Methods section:

*“For any positive findings, we ran colocalisation analysis using the ‘coloc’ package in R [3], to test if there was violation of the MR exclusion restriction assumption. This can be generated through instrument-exposure and instrument-outcome associations, driven by distinct causal variants that are in LD with each other. The ‘coloc’ package in R enumerates every possible configuration of causal variants for two traits, calculating the support for that causal model in the form of a Bayes factor, assuming that at most one causal variant per trait exists in the region and there are similar LD structures across the two samples. Approximate Bayes factor colocalisation analysis fine maps each trait under a single causal variant assumption and then integrates these over two posterior distributions to estimate probabilities that those variants are shared [3]. A posterior probability of ≥ 0.80 is considered evidence to support a particular configuration tested in ‘coloc’. Using genomic regions of 1 Mb either side of the lead variant for the genes of interest, we investigated whether findings reflect shared causal variants.” (lines 327-339)*

The following has also been added to our Results section:

*“To further investigate whether the positive findings for PCSK9 and LDLR are due to violation of the exclusion restriction assumption, colocalisation analysis was carried out [3]. This showed no conclusive evidence of shared causal variants between LDL-C and oral/oropharyngeal cancer, with posterior probabilities of 0.055 for PCSK9 and 0.026 for LDLR, respectively (****S12 Table****).” (line 474-478)*

The following has also been added to our Discussion section:

*“Further colocalisation analysis for PCSK9 and LDLR showed no conclusive evidence of shared causal variants between LDL-C and oral and oropharyngeal cancer; however, the outcome dataset had a relatively smaller sample size, and typically large sample sizes are required across both traits for better accuracy (****S12 Table****) [3, 4].” (lines 517-520)*

**3) In the Discussion, the authors nicely discuss the potential association between PCSK9-alcohol and head & neck cancer. A multivariable MR analysis adjusting for alcohol is warranted, although power may be low.**

The positive effect we observed between proxied *PCSK9* inhibition and oral/ oropharyngeal cancer was the opposite to what would be anticipated. Based on previous studies a protective effect of *PCSK9* inhibition would be expected, given that elevated *PCSK9* expression was previously associated with alcohol use disorder and alcohol is a well-established risk for head and neck cancer. In addition to this, given the nuances of multivariable MR (and length of the current paper with primary and secondary analyses), we feel this would be better suited to subsequent analysis and perhaps a separate publication depending on the findings.

We have however, added more detail to the text in the Discussion below:

*“Further investigation is required to untangle the relationship between PCSK9, alcohol and head and neck cancer, including a subsequent multivariable MR analysis”. (lines 571-573)*

**4) The authors made a comment in lines 553-555 about potential for immortal time bias in observational studies, but this bias is far more prevalent in studies of cancer survival than incidence.**

We accept the reviewer’s comment about immortal time bias and have changed the wording of our statement to “may have”, in order to reflect this. We are trying here to convey that the longer people survive without cancer, the more likely they are to be prescribed statins and this might be why it appears as though statins are having a protective effect, when in reality it could be another confounder.

*In addition to confounding, immortal time bias may have inflated observational results because, to be classified as a long-term statin user necessitates that users survived without cancer over a long period [5, 6] (lines 579-581)* **5) The last sentence of the Conclusion seems unsubstantiated based on the findings of the current literature and the current paper.**

We agree with this point and have now removed the final sentence of our Conclusion.

A small number of additional minor edits have also been made, as highlighted in the main text.

**References**

1.	Kao LT, Hung SH, Kao PF, Liu JC, Lin HC. Inverse association between statin use and head and neck cancer: Population-based case-control study in Han population. Head Neck-J Sci Spec. 2019;41(5):1193-8.

2.	Zhang Y, Hedo R, Rivera A, Rull R, Richardson S, Tu XM. Post hoc power analysis: is it an informative and meaningful analysis? General Psychiatry. 2019;32(4):e100069.

3.	Wallace C. Eliciting priors and relaxing the single causal variant assumption in colocalisation analyses. PLOS Genetics. 2020;16(4):e1008720.

4.	Ni G, Moser G, Ripke S, Neale BM, Corvin A, Walters JTR, et al. Estimation of Genetic Correlation via Linkage Disequilibrium Score Regression and Genomic Restricted Maximum Likelihood. The American Journal of Human Genetics. 2018;102(6):1185-94.

5.	Dickerman BA, Garcia-Albeniz X, Logan RW, Denaxas S, Hernan MA. Avoidable flaws in observational analyses: an application to statins and cancer. Nat Med. 2019;25(10):1601-6.

6.	Hernan MA, Sauer BC, Hernandez-Diaz S, Platt R, Shrier I. Specifying a target trial prevents immortal time bias and other self-inflicted injuries in observational analyses. J Clin Epidemiol. 2016;79:70-5.
